# IL7R^+^ T Cell‐Macrophage Crosstalk Links Asthma to Alzheimer's Pathogenesis: Integrating Mendelian Randomization and CellChat Analysis

**DOI:** 10.1002/brb3.70809

**Published:** 2025-09-15

**Authors:** Jing Yang, Zixing Liu, Xiaofang Li, Yichong Qiu, Qiong Liu, Xiufang Huang, Leshen Lian

**Affiliations:** ^1^ The First Affiliated Hospital of Guangzhou University of Chinese Medicine Guangzhou Guangdong China; ^2^ Lingnan Medical Research Center of Guangzhou University of Chinese Medicine Guangzhou Guangdong China; ^3^ Wuhan Hospital of Traditional Chinese Medicine Wuhan Hubei China; ^4^ Dongguan Hospital Affiliated to Guangzhou University of Chinese Medicine Dongguan China

**Keywords:** Alzheimer's disease, inflammatory factor, Mendelian randomization, severe asthma, single‐cell RNA sequencing analysis

## Abstract

**Purpose:**

Epidemiological investigation has revealed a higher incidence of Alzheimer's disease (AD) in individuals with severe asthma. However, the causality of this relationship remains uncertain. The current research aimed to examine the potential link between genetically predicted moderate to severe asthma and the risk of AD using Mendelian randomization (MR) analysis.

**Methods:**

Summary statistics obtained from genome‐wide association studies of AD (*n* = 455,258) and moderate to severe asthma (*n* = 57,695) in individuals of European ancestry were utilized in this MR study. SMR analysis was also performed to investigate whether the expression of these genes was correlated with AD or moderate to severe asthma outcomes to detect a causal relationship between moderate to severe asthma and AD. Genome‐wide genetic correlation between moderate to severe asthma and AD was estimated using linkage disequilibrium score regression (LDSC). Single‐cell RNA sequencing (scRNA‐seq) datasets of asthma‐related peripheral blood mononuclear cell (PBMC) data and AD cerebrospinal fluid (CSF) were obtained to further investigate the crosstalk between the different biological pathways in asthma and AD.

**Results:**

The impact of moderate to severe asthma on AD risk persisted (OR_IVW _= 1.01, 95% CI = 1.00–1.02, *p* = 3.85 × 10^−3^) after controlling for confounder risk factors in multivariable MR analyses. Additionally, the study showed that 1.8% of the total effect (moderate to severe asthma) was mediated by eosinophils. SMR analysis and the gene‐wide MR analysis revealed numerous gene targets linked to the susceptibility of AD and moderate to severe asthma. Among these targets, FPR1 (Formyl Peptide Receptor 1), IL1RAP (Interleukin 1 Receptor Accessory Protein), IL7R (Interleukin 7 Receptor), and IL18RAP (Interleukin 18 Receptor Accessory Protein) warrant additional exploration as potential therapeutic targets for AD and moderate to severe asthma. LDSC analysis revealed no significant overlap between asthma and AD (rg = 0.0436, SE = 0.0813, *p* = 0.592), suggesting distinct genetic architectures. Integrated single‐cell RNA sequencing analysis of asthma PBMCs and AD CSF revealed IL7R may utilize the MIF‐CD74‐CXCR4 pathway to complete crosstalk between CD4 T cells and macrophages and contribute to AD disease development.

## Graphical abstract

β1 represents the regression coefficients for the association between moderate to severe asthma and mediators; β2 represents the regression coefficients for the association between mediators and Alzheimer's disease; β3 represents the total effect between moderate to severe asthma and Alzheimer's disease, without the adjustment for mediators.

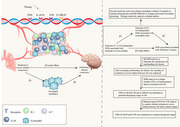

**Abbreviations**
AD: Alzheimer's disease
CI: confidence interval
CSF: cerebrospinal fluid
eQTL: expression quantitative trait loci
FPR1: formyl peptide receptor 1
GWAS: genome‐wide association study
HEIDI: heterogeneity in dependent instruments
IL18RAP: interleukin 18 receptor accessory protein
IL1RAP: interleukin 1 receptor accessory protein
IL7R: interleukin 7 receptor
IVW: inverse variance weighted
LD: linkage disequilibrium
LDSC: linkage disequilibrium score regression
MAF: minor allele frequency
MR: Mendelian randomization
MR‐PRESSO: MR pleiotropy residual sum and outlier
MVMR: multivariable MR
OR: odds ratio
PBMC: peripheral blood mononuclear cell
PCA: principal component analysis
PRESSO: pleiotropy residuals and outliers
scRNA‐seq: single‐cell RNA sequencing
scRNA‐seq: single‐cell RNA sequencing
SMR: summary‐data‐based MR
SNPs: single‐nucleotide Polymorphisms
UMAP: Uniform Manifold Approximation and Projection

## 1 Introduction

Alzheimer's disease (AD) is the most common type of dementia (Ballard et al. 2011), which impacts at least 27 million individuals and corresponds to 60% to 70% of all dementia cases (Ferreira et al. 2014). AD usually involves memory loss of recent events and is characterized by a decline in cognition, function, and behavior (Scheltens et al. 2021). The occurrence of AD also brings a huge impact on the life of the patient's family, including mental stresses, negative emotional influences, and an unsustainable burden to society Alzheimer's Association (2020). The number of AD cases is steadily increasing, and with the aging population, the global number of dementia patients is expected to reach 152 million by 2050 unless effective therapy or prevention is available (Patterson 2018).

Clearly, AD is a major health problem all over the world. It has been estimated that 35% of the total risk factors of AD can be modified, indicating that placing more emphasis on these factors could prevent up to one‐third of all cases (Grøntvedt et al. 2018). These factors could be classified into several groups, such as social psychology, preexisting medical conditions, lifestyle choices, and others, and they can lead to a better understanding of the potential impact on cognition and aid in the prevention of AD.

Chronic illnesses in the elderly can deteriorate cognitive abilities, functional status, and lead to depression in AD patients. This trend has been noted in numerous cases of cardiac insufficiency, metabolic ailments like diabetes, malignant diseases, and others (Weiner et al. 2010; Alzheimer's Association^2009^; Cukierman‐Yaffe et al. 2009). Meanwhile, epidemiological studies have shown that AD occurs more frequently in asthmatics, especially those with severe asthma (Rosenkranz et al. 2022), which could potentially increase their risk of neural damage and cognitive impairment. Previous research has indicated a correlation between asthma and dementia, but the findings remain inconclusive (Eriksson et al. 2008; Jelicic and Kempen 1997; Ng et al. 2007). There are few studies on the enduring effect of moderate to severe asthma on cognitive abilities or AD. Based on this, we employed Mendelian randomization (MR) and single‐cell RNA sequencing (scRNA‐seq) analysis to estimate the effect of moderate to severe asthma on AD.

MR is a technique that uses genetic data to establish causal correlations between exposure factors and disease. It operates on the basis of Mendelian laws of inheritance, according to which genes are allocated to progeny at random during the development of germ cells. Due to random assignment, genetic variants that are highly correlated with exposure factors—which are unaffected by other confounders—are chosen as instrumental variables. Therefore, the use of MR can eradicate the effect of confounders and steer clear of reverse causation and other inaccuracies often encountered in observational epidemiological studies (Ebrahim and Smith 2008). To establish the causation of relevant risk factors for certain conditions (Smith and Ebrahim 2003), the MR chooses instrumental exposure variables; a causal effect between the exposure factor and the disease can be inferred if a relationship between the instrumental variable and the disease is discovered.

Thus, this study utilized two‐sample MR to reveal the causal relationship between moderate to severe asthma and AD. Further, we used a two‐step MR approach to explore whether these common risk factors act as mediators for the effects of moderate to severe asthma. The multivariable MR method (MVMR) was conducted to evaluate the effect of moderate to severe asthma on AD after adjusting for the confounders. The summary‐data‐based MR (SMR) method was utilized to explore the potential therapeutic targets for moderate to severe asthma with AD. ScRNA‐seq analysis of asthma PBMCs and AD CSF to detect the pathogenic pathway.

## 2 Methods

### 2.1 Study Ethics

The two‐sample MR study utilized publicly available genome‐wide association study (GWAS) and expression quantitative trait loci (eQTLs) research (Table S1). Therefore, no further ethics approval was required.

### 2.2 Selection of Genetic Instruments of MR

In two‐sample MR and MVMR, single‐nucleotide polymorphisms (SNPs) among the genome‐wide significant SNPs (*p *< 5 × 10^−8^) were identified in GWAS. Genetic variants with uncertain strand codification (A/T or C/G) were excluded. SNPs that had a linkage disequilibrium (LD) level of *r* ^2^ < 0.001 and LD distance > 10,000 kb were ultimately removed. Next, to evaluate the strength of instrumental variables, we calculated *F*‐statistics (Palmer et al. 2012) that are relevant to the explained variance of exposure (*R* ^2^), sample size (*n*), and the number of SNPs (*k*) by the formula F=[(n−k−1)/k]/[R2/(1−R2)](Burgess and Thompson 2011). In general, *F* > 10 demonstrates that SNPs could predict well. Table S4 provided a comprehensive list of the selected SNPs for 15 moderate to severe asthma and 451 eosinophil counts in this MR study.

### 2.3 GWAS Outcome Source

GWAS outcome data was obtained from a genome‐wide meta‐analysis study. The study population consisted of 455,258 individuals of European ancestry in a meta‐analysis in three phases containing up to four independent consortia. In the meta‐analysis, there were 455,258 participants in total, consisting of 71,880 cases and 383,378 controls. All information on the GWAS datasets is shown in Table S1. Association statistics of AD and moderate to severe asthma GWAS were listed in Table S2 and Table S3.

### 2.4 MR Statistical Analysis

In this study, the principal approach for conducting two‐sample MR was inverse variance weighting (IVW) (Ehret et al. 2011), followed by MR Egger regression and weighted median (Bowden et al. 2016). The IVW approach was used to demonstrate the causal relationship between exposure and outcome the results were presented as odds ratios (OR) and 95% confidence intervals (CI). IVW is widely used as the main analysis method because it has the highest statistical power under the assumption of no horizontal pleiotropy. IVW integrates the effects of all instrumental variables through a weighted regression model to provide unbiased causal estimates. The IVW method provides unbiased causal estimates under two core assumptions: (1) All genetic instruments satisfy MR validity criteria (relevance, independence from confounders, and exclusion restriction), and (2) balanced horizontal pleiotropy (pleiotropic effects have a mean of zero and are uncorrelated with instrument strength). Violations are evaluated through Cochran's *Q* test and funnel plot (heterogeneity and pleiotropic bias). Consistency checks using robust MR methods (e.g., MR‐Egger, weighted median) when IVW assumptions fail. The MR‐Egger regression was applied if there was notable horizontal pleiotropy; otherwise, the IVW method was employed. However, to ensure the robustness of the results, we also used MR‐Egger regression, weighted median, and MR‐PRESSO (pleiotropy residuals and outliers) as sensitivity analysis methods to assess the potential pleiotropy and the impact of outliers.

We further utilized several sensitivity tests, including the MR Egger analysis for detecting and adjusting for pleiotropy, specifically, that genetic variants are robustly associated with the exposure (instrument strength) and lack unbalanced confounding (Burgess and Thompson 2017). Horizontal pleiotropy is inferred if the MR‐Egger intercept deviates significantly from zero (*p* for intercept < 0.05), indicating directional pleiotropy. Funnel plots should accompany MR‐Egger to evaluate heterogeneity and validate model assumptions. MR‐PRESSO was also utilized to evaluate and rectify horizontal pleiotropy (Ong and MacGregor 2019). MR‐PRESSO identifies and corrects horizontal pleiotropic outliers through a three‐step process: (1) Global Test of Pleiotropy: A residual sum of squares comparison between observed and expected (under the null hypothesis of no pleiotropy) genetic variant associations detects overall pleiotropy (*p* < 0.05); (2) Outlier Detection: Iterative removal of individual variants followed by RSS comparison identifies specific pleiotropic outliers (*p* < 0.05); (3) Distortion Correction: The causal effect is reestimated after removing outliers, and a distortion test evaluates whether the outlier‐corrected estimate significantly differs from the original estimate (*p* < 0.05). When the proportion of horizontal pleiotropy variants is less than 10%, MR‐PRESSO exhibits superior precision and is less biased and has better precision compared to IVW and MR‐Egger (Verbanck et al. 2018). To assess whether a single SNP was driven or had an impact on the MR analysis results, a leave‐one‐out examination was conducted.

MVMR is an extension of univariable MR that takes pleiotropy among multiple traits into account. We estimated and measured the impact of mediators, such as eosinophil counts, on the relationship between moderate‐to‐severe asthma and AD using two‐step MR and MVMR. The two‐step approach greatly reduces the biases inherent in the typical multivariable approach (Richmond et al. 2016). In MVMR, the effect of exposure was decomposed into direct and indirect effects. In MVMR models, covariates refer to secondary exposures (BMI, diabetes, hypertension, cigarettes smoked per day, and total cholesterol) that may exhibit pleiotropic effects on both the primary exposure (moderate to severe asthma) and the outcome (AD). By simultaneously regressing genetic associations with the outcome on genetic associations with all exposures, MVMR estimates the direct causal effect of each exposure, adjusted for pleiotropy via other exposures. This distinguishes MVMR covariates from conventional confounders. The graphical abstract displayed a visual representation of the analysis processes. The percent mediation was computed using the formula proportionmediated=(β1×β2)/(β3)] (*β*1 = exposure‐mediator effect, *β*2 = mediator‐outcome effect, *β*3 = total effect). In our study, *β*1 is the effect of moderate to severe asthma‐eosinophils, *β*2 is the effect of eosinophils‐AD, and *β*3 is the effect of moderate to severe asthma‐AD. Version 0.5.6 of TwoSampleMR was utilized for MR analysis in the R package (version 4.2.1). Ultimately, to evaluate the possibility of latent pleiotropy, we conducted bidirectional Mendelian randomization, also referred to as reverse MR, for moderate to severe asthma and AD (Zheng et al. 2017).

### 2.5 SMR Statistical Analysis

The identification of eQTLs, or genetic variants that impact gene expression, has become the subject of an increasing number of studies (GTEx Consortium 2020; Kerimov et al. 2021). In order to identify potential causal genetic variants for AD, an SMR analysis of GWAS with the whole blood eQTLs was conducted. SMR software, version 1.03 (https://cnsgenomics.com/software/smr/Overview), was utilized for allele harmonization and examination. Blood‐derived *cis*‐eQTL summary statistics were obtained from the eQTLGen Consortium Phase I (31,684 individuals; 37 cohorts) (Võsa et al. 2021) (https://www.eqtlgen.org/). Cohorts were exclusively of European ancestry, and technical confounders (e.g., cell counts, batch effects) were pre‐adjusted. The workflow proceeds as follows: (1) Instrument Selection: Genome‐wide significant SNPs (*p* < 5 × 10^−8^) are selected as instrumental variables, which included only SNPs with MAF greater than 1% (Table 1); (2) *cis*‐eQTL Colocalization: For each candidate gene, the top *cis*‐eQTL (within ±1 Mb of the gene transcription start site) at the GWAS locus is tested for LD‐adjusted association with the trait; (3) Pleiotropy Adjustment: the HEIDI (Heterogeneity in Dependent Instruments) test (Zhu et al. 2016) distinguishes true pleiotropy (same causal variant for both trait and expression) from linkage (distinct causal variants). A gene‐trait association is robust if the SMR test reaches significance (*p* < 0.01) and the HEIDI test supports pleiotropy (HEIDI *p* > 0.01) (Chauquet et al. 2021). Analyses require harmonized GWAS and eQTL summary statistics from matched populations.

**TABLE 1:** Information on genetic instruments.

Exposure	Genetic instruments	
	Genetic variants associated with mRNA expression levels (eQTLs)	Genetic variants associated with inflammatory factor level
FPR1	12 common *cis*‐eQTLs (MAF >1%) in blood for the FPR1 gene (*p* < 5.0 × 10^−8^), top SNP: rs6509570	7 common SNPs (MAF > 1%) in low linkage disequilibrium (*r* ^2^ < 0.30), associated with eosinophil cell count (*p* < 5.0 × 10^−8^), located within ±100 kb windows from the FPR1 region
IL1RAP	3 common *cis*‐eQTLs (MAF > 1%) in blood for the IL1RAP gene (*p* < 5.0 × 10^−8^), top SNP: rs78888631	12 common SNPs (MAF > 1%) in low linkage disequilibrium (*r* ^2^ < 0.30), associated with Interleukin‐1 Receptor accessory protein (*p* < 5.0 × 10^−8^), located within ±100 kb windows from the IL1RAP region
IL7R	11 common *cis*‐eQTLs (MAF > 1%) in blood for the IL7R gene (*p* < 5.0 × 10^−8^), top SNP: rs10058453	3 common SNPs (MAF > 1%) in low linkage disequilibrium (*r* ^2^ < 0.30), associated with Interleukin‐7 receptor subunit alpha (*p* < 5.0 × 10^−8^), located within ±100 kb windows from the IL7R region
IL18RAP	44 common *cis*‐eQTLs (MAF > 1%) in blood for IL18RAP gene (*p* < 5.0 × 10^−8^), top SNP: rs6734762	3 common SNPs (MAF > 1%) in low linkage disequilibrium (*r* ^2^ < 0.30), associated with Interleukin‐18 receptor 1 (*p* < 5.0 × 10^−8^), located within ±100 kb windows from IL18RAP region
Statistical analyses		
Primary analysis	Summary‐data‐based Mendelian randomization	Inverse‐variance‐weighted Mendelian randomization

Abbreviations: eQTLs, expression quantitative trait loci; GWAS, genome‐wide association study; HEIDI, heterogeneity in dependent instruments; IVW‐MR, inverse‐variance‐weighted Mendelian randomization; MAF, minor allele frequency; SMR, summary‐data‐based Mendelian randomization; SNP, single‐nucleotide polymorphism.

Available eQTLs for drugs targeting genes that served as a proxy for exposure to each inflammatory factor were demonstrated in Table 1.

### 2.6 Linkage Disequilibrium Score Regression

To assess the shared genetic architecture between moderate‐to‐severe asthma and AD, genome‐wide genetic correlation was estimated using linkage disequilibrium score regression (LDSC). Summary statistics from previously published GWAS of both traits were harmonized to remove strand‐ambiguous SNPs and aligned to the human reference genome (GRCh37/hg19). Precomputed LD scores derived from the European ancestry subset of the 1000 Genomes Project Phase 3 were utilized to model the relationship between SNP effect sizes and LD patterns. Genetic correlation coefficients (rg) were calculated using the LDSC framework (v1.0.1) with default parameters, including intercept adjustment to account for potential confounding factors such as cryptic sample overlap, population stratification, and inflation of test statistics due to residual polygenicity (Bulik‐Sullivan et al. 2015). Significance of genetic correlation was determined via a two‐tailed *Z*‐test, and Bonferroni correction was applied to adjust for multiple comparisons across trait pairs. The analysis further partitioned heritability to quantify the contribution of functional genomic annotations (e.g., conserved regions, enhancers) to the observed genetic overlap.

### 2.7 scRNA‐seq Data Processing and Analysis

scRNA‐seq datasets were obtained from publicly available repositories. Asthma‐related PBMCs data were sourced from the GEO accession GSE172495 (A. Chen et al. 2021), including five asthma samples (GSM5257967–GSM5257971) and five healthy PBMC controls (GSE163668: GSM4995433, GSM4995441, GSM4995448, GSM4995450, GSM4995456) (Combes et al. 2021). AD and control samples were acquired from GSE134577 (Oh et al. 2021), comprising four AD brain tissue samples (GSM3984200, GSM3984204, GSM3984206, GSM3984213) and four normal CSF controls (GSM3984199, GSM3984202, GSM3984209, GSM3984216).

This study conducted a systematic analysis of scRNA‐seq data, integrating AD brain tissue, CSF samples, and asthma patient PBMCs datasets. The raw data were processed using the Seurat pipeline: cross‐tissue data integration was achieved through sample identifier mapping (e.g., AD samples labeled as ASD.s1‐4, normal CSF labeled as CSF.s1‐4), followed by rigorous quality control (retaining cells with 200–4,000 detected genes, mitochondrial gene percentage < 10%, and hemoglobin gene filtering to exclude erythrocyte contamination). Data normalization was performed using the LogNormalize method (scaling factor = 10,000), and 2000 highly variable genes were selected via variance stabilizing transformation (vst) for principal component analysis (PCA). To address batch effects from tissue sources (PBMC vs. brain/CSF), the Harmony algorithm (parameters: theta = 3, lambda = 0.8, max.iter = 50) was applied for integration optimization, resulting in a cross‐disease single‐cell transcriptomic atlas. Uniform Manifold Approximation and Projection (UMAP) was applied for dimensionality reduction using the first 15 harmony‐corrected principal components. Clustering was performed at a resolution of 0.5, and cell types were annotated via clustifyR with manual refinement based on canonical markers from the CellMarker and PanglaoDB databases (Fu et al. 2020). This pipeline established a high‐quality data foundation for subsequent cell‐type‐specific communication analyses, such as IL7R^+^ T cell‐macrophage interactions.

Cell‐type proportions were quantified across the asthma and AD cohorts. Expression patterns of FPR1, IL1RAP, IL7R, and IL18RAP were visualized using violin plots, density maps (Nebulosa package), and bubble plots. CD4 T cells were stratified into IL7R^+^ and IL7R^−^ subsets based on normalized expression counts (IL7R^+^: counts > 0). To investigate intercellular communication, a subset of 3000 cells was randomly sampled for analysis using CellChat (Jin et al. 2021). Ligand–receptor interactions from the secreted signaling and cell–cell contact databases were prioritized, and communication probabilities were computed with population size adjustment. Pathway enrichment analysis identified biologically relevant signaling axes, and significant interactions were filtered (*p *< 0.05 after Bonferroni correction).

## 3 Result

### 3.1 Genetic Instruments Selection

The *F*‐statistics for genetic instruments of drug targets and several exposures were over 10, indicating that our study was unlikely subject to weak instrument bias. The instrument variants of the drug target gene were identified from eQTLGen, and the most significant *cis*‐eQTL SNP was chosen as a genetic instrument for the target gene of each drug.

### 3.2 MR Causal Analysis

The present study's MR findings were consistent with a prior investigation (Rosenkranz et al. 2022), indicating a significant association between moderate to severe asthma and the risk of AD (OR_IVW _= 1.01, 95% CI = 1.00–1.02, *p *= 3.85 × 10^−3^). Unbiased estimates for causality were reflected by both IVW and MR Egger, indicating the absence of pleiotropy. The relationship between weighted median MR (OR = 1.01, 95% CI = 1.00–1.02, *p *= 0.171) and MR Egger (OR = 1.03, 95% CI = 0.98–1.08, *p *= 0.293) was generally in agreement with IVW MR, but the intervals of confidence were broader because of reduced statistical power (Verbanck et al. 2018). The effect estimates for all methods were in the same direction, although not significant, but this consistency suggests that no obvious bias (such as pleiotropy) confounded the results, as the conclusions of the different methods did not conflict. Even if some instrumental variables are invalid, the weighted median can still provide unbiased estimates. Its nonsignificant results further support the conclusion of IVW. Individual SNPs were estimated without statistically significant horizontal pleiotropy and heterogeneity, as shown by the MR Egger intercept tests and the Cochran's *Q* test (*p* intercept = 0.539, *Q p* value = 0.359). Further analysis in MR‐PRESSO also revealed an identical result (*p* value of the global heterogeneity test = 0.385). All the results above indicated that the potential horizontal pleiotropy of the SNPs did not influence our results. These results were depicted in detail in Table 2.

**TABLE 2:** Two‐step MR association between moderate to severe asthma mediated by eosinophil counts and AD.

Exposure	Outcome	Method	Number of SNPs	beta	SE	OR	95%CI	*p* value	*p*‐value for Cochran's Q test	*p* value for the MR‐Egger intercept	*p* value for MR‐PRESSO Global test
Moderate to severe asthma	Alzheimer's disease	MR‐Egger	15	0.029	0.026	1.03	0.98–1.08	0.292	0.318	0.539	0.385
		Weighted median		0.008	0.006	1.01	1.00–1.02	0.171	0.359		
		Inverse variance weighted		0.012	0.004	1.01	1.00–1.02	0.004			
Moderate to severe asthma	Eosinophil counts	MR‐Egger	4	0.233	0.148	1.26	0.95–1.69	0.255	3.06E‐05	0.511	0.100
		Weighted median		0.106	0.015	1.11	1.08–1.15	1.06E‐12	5.06E‐06		
		Inverse variance weighted		0.117	0.018	1.12	1.08–1.17	1.89E‐10			
Eosinophil counts	Alzheimer's disease	MR‐Egger	451	0.039	0.017	1.04	1.00–1.07	2.10E‐02	9.87E‐06	0.152	<1e‐04
		Weighted median		0.009	0.012	1.01	0.99–1.03	4.29E‐01	8.12E‐06		
		Inverse variance weighted		0.018	0.008	1.02	1.00–1.03	2.80E‐02			

**FIGURE 1:**
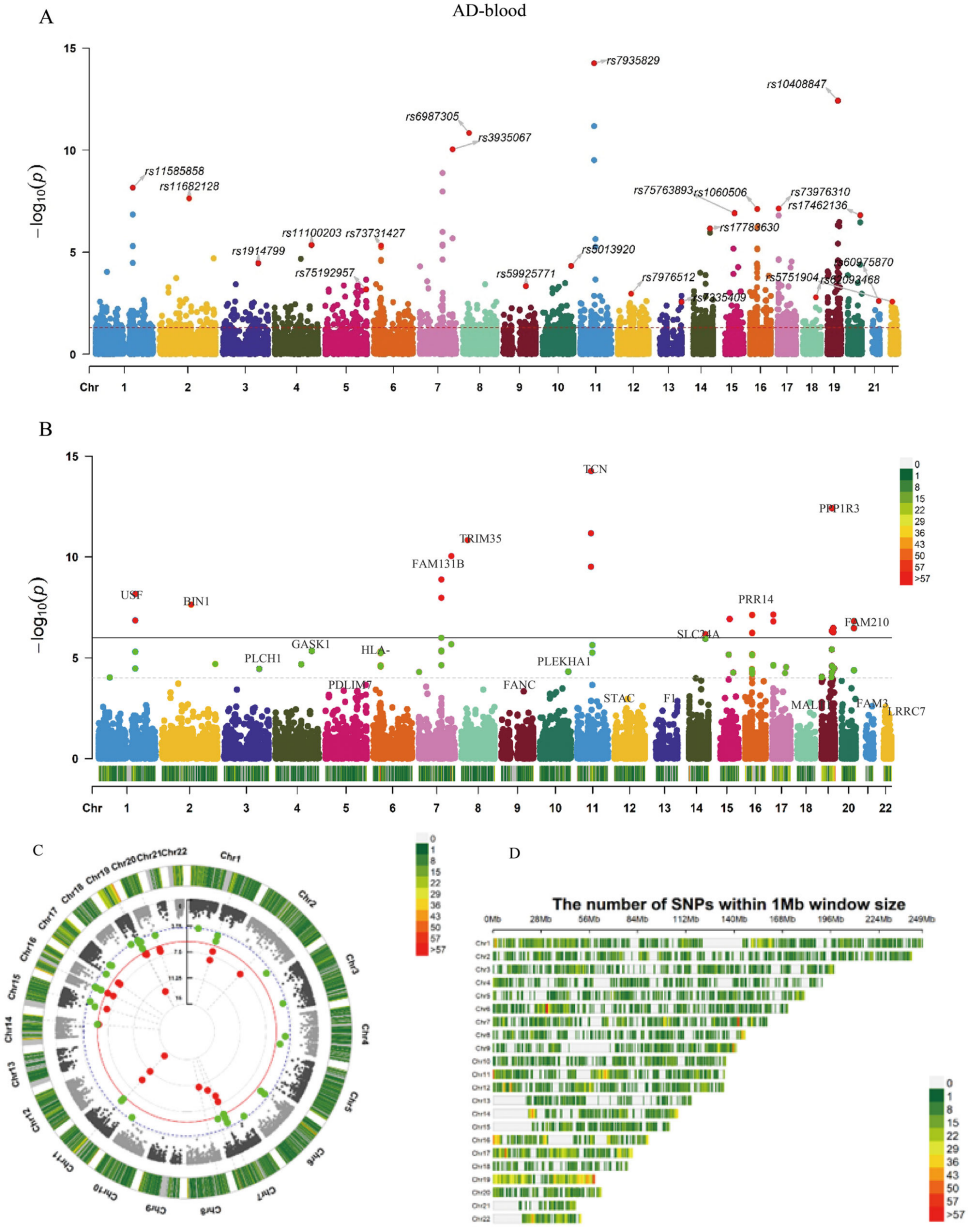
Genome‐wide significant loci and MR analysis for moderate to severe asthma and Alzheimer's disease. (A) genome‐wide significant loci for moderate to severe asthma; (B) genome‐wide significant loci for AD; (C) funnel plot of two‐sample MR for moderate to severe asthma between AD; (D) scatter plot of two‐sample MR for moderate to severe asthma between AD; (E) leave‐one‐out plot of two‐sample MR for moderate to severe asthma between AD.

In the leave‐one‐out sensitivity analysis, none of the SNPs significantly compromised the overall effect of moderate to severe asthma on AD. In addition, the funnel plot was approximately symmetrical, suggesting no pleiotropy. Detailed visible results were shown in Figure 1.

After univariable MR analysis, MVMR analysis adjusted for potential confounders was performed. In keeping with the previous studies (Silva et al. 2019; Crous‐Bou et al. 2017; Zhang et al. 2021; Pasqualetti et al. 2022), we considered smoking, BMI, hypertension, diabetes, and total cholesterol as strong risk factors of AD. After the confounding factors were excluded, there still existed a causality between moderate to severe asthma and AD after excluding these confounders (OR_IVW _= 1.04, 95% CI = 1.01–1.07, *p *= 4.20 × 10^−3^) (Table S5). The reverse MR study seeks to explore whether there is no reverse causal link between moderate to severe asthma and AD (Table S6).

### 3.3 MR Causal Mediation Analysis

In two‐step MR, first, we observed evidence for association between moderate to severe asthma and eosinophil counts (OR_IVW _= 1.12, 95% CI = 1.08–1.17, *p *= 1.89×10^−10^). Then the MR results supported an association between eosinophil counts and AD (OR_IVW _= 1.02, 95% CI = 1.00–1.03, *p *= 0.033). The aforementioned findings establish that eosinophil counts partially mediate the association between moderate to severe asthma and AD.

To further analyze the mediating effect, we calculated by formula that eosinophil count mediated 1.8% of the total effect of moderate to severe asthma on AD. In the heterogeneity test and the MR‐PRESSO test, we found significant heterogeneity for our outcomes. To assess the causal estimate from IVW, the MR‐PRESSO distortion test was used before and after excluding the horizontal pleiotropic outlier variants due to the existence of heterogeneity. The results of the MR‐PRESSO distortion test proved that the causal estimate of our study was not distorted by horizontal pleiotropic outlier variants (Verbanck et al. 2018) (*p *= NA). It can be seen that our results were not influenced by heterogeneity. Detailed MR analysis results were presented in Table 2.

### 3.4 SMR Analysis

**FIGURE 2:**
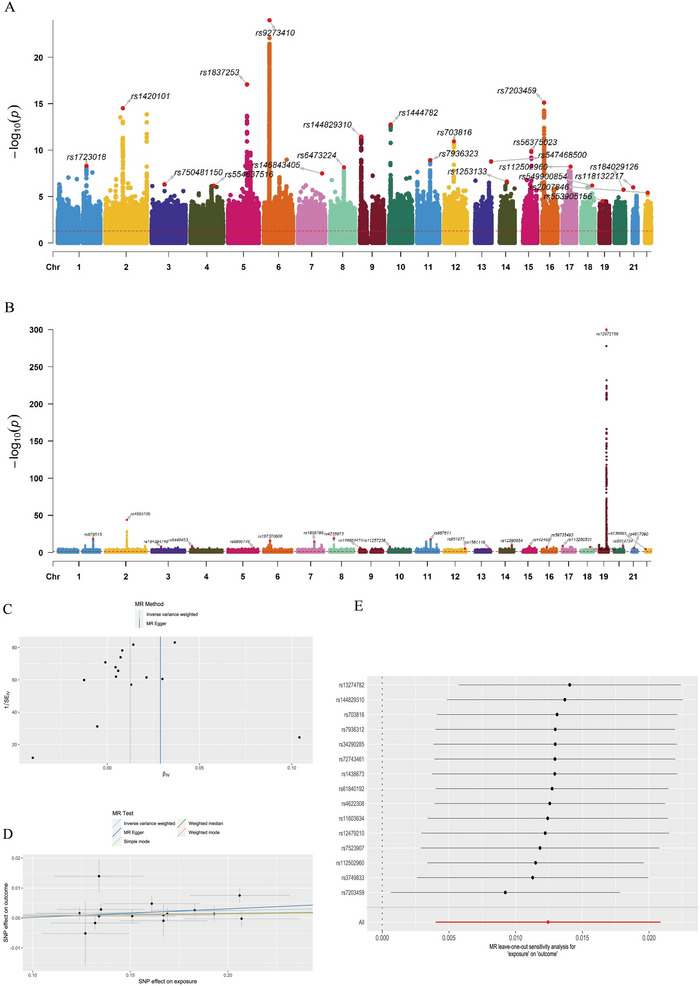
SMR analysis of asthma and Alzheimer's disease. (A) Manhattan plot of the SMR analysis of GWAS for AD and eQTL for whole blood with top SNP for each chromosome; (B) Manhattan plot of the SMR analysis of GWAS for AD and eQTL for whole blood with top gene for each chromosome; (C) Donut chart of the SMR analysis of GWAS for AD and eQTL for whole blood with top gene for each chromosome; (D) SNP density plot of the SMR analysis of GWAS for AD and eQTL for whole blood with top gene for each chromosome.

We selected 11 SNPs in FPR1 (Formyl Peptide Receptor 1), 25 SNPs in IL1RAP (Interleukin 1 Receptor Accessory Protein), 5 SNPs in IL7R (Interleukin 7 Receptor), and 15 SNPs in IL18RAP (Interleukin 18 Receptor Accessory Protein) (Table S4). Suggestive evidence for the correlation between elevated levels of inflammatory factors in blood was discovered through SMR analysis (Table S5). In concert, the result of SMR analysis of GWAS with the whole blood eQTLs was summarized in the form of pictures (Figure 2).

The HEIDI test indicated that none of the observed associations were a result of linkage (*p *> 0.01) during SMR analysis. Additionally, we conducted further analyses to assess the existence of horizontal pleiotropy in the relationship between inflammatory factor expression and AD outcomes. Specifically, we investigated whether there was a correlation between the expression of additional genes in the vicinity significantly associated with the top eQTL SNP (instrument variant) of inflammatory factors and AD. We identified other genes with top SNPs whose expression was associated with the instrument variant (Table 6). Only four genes have available eQTLs at a genome‐wide significance level (*p *< 5.0 × 10^−8^). Out of the four genes, only FPR1 expression had a significant correlation with AD, indicating a minor involvement of horizontal pleiotropy in the observed connections.

In the analysis of IVW‐MR, no heterogeneity was observed in all reported results according to the Cochran *Q* test (all *p *> 0.05; Tables S7 and S8). Neither the intercept term in MR‐Egger regression nor the MR‐PRESSO analysis indicated any significant overall horizontal pleiotropy (all *p *> 0.05).

### 3.5 LDSC Results

The SNP‐based heritability of moderate‐to‐severe asthma was estimated at 8.06% (standard error [SE] = 0.0114, *Z* = 7.06, *p* = 1.61 × 10^−12^), indicating a significant contribution of common genetic variants to disease risk. In contrast, AD exhibited a lower but statistically significant heritability of 1.00% (SE = 0.00181, *Z* = 5.55, *p* = 2.93 × 10^−8^). Both traits showed minimal genomic inflation (λGC = 1.09 for asthma and AD), consistent with negligible confounding from population stratification or technical artifacts. Genetic correlation analysis revealed no significant overlap between asthma and AD (rg = 0.0436, SE = 0.0813, *p* = 0.592), suggesting distinct genetic architectures. Partitioned heritability analysis further highlighted disease‐specific enrichment patterns: asthma heritability was disproportionately driven by conserved genomic regions and immune‐related enhancers (enrichment *p* < 0.05), whereas AD heritability clustered in neuronal chromatin states, including histone H3K4me3 marks in the prefrontal cortex *(p* < 0.01). Intermediate matrices (e.g., variance–covariance and signal matrices) are provided in Table S12.

### 3.6 Cross‐Tissue Single‐Cell Profiling Reveals Disease‐Specific Immune Landscapes in Asthma (PBMC) and AD (CSF)

**FIGURE 3:**
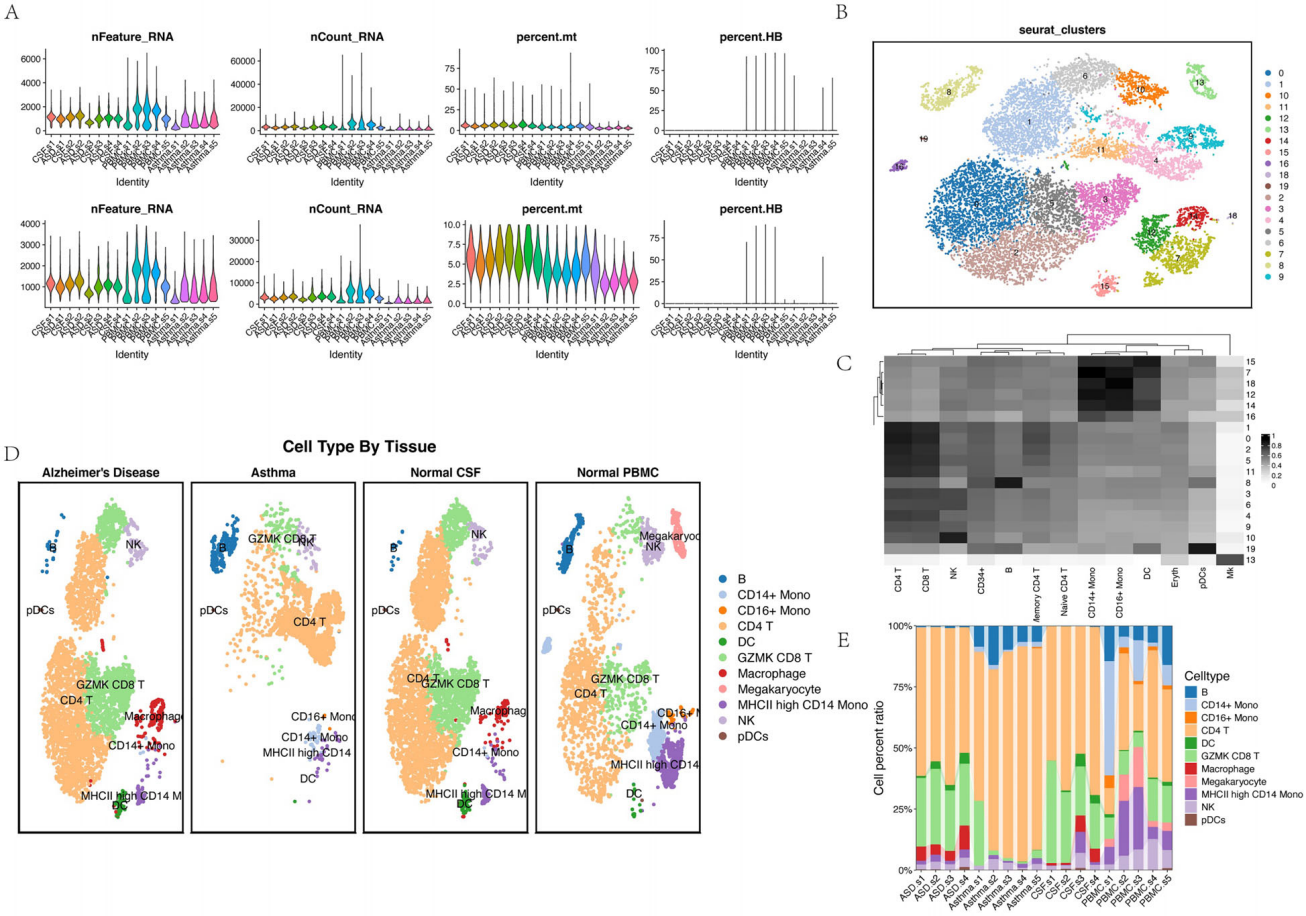
Cross‐tissue single‐cell profiling reveals disease‐specific immune landscapes in asthma (PBMC) and Alzheimer's disease (CSF). (A) Quality control metrics: Violin plots of genes/cell (nFeature_RNA), counts/cell (nCount_RNA), mitochondrial (percent.mt), and hemoglobin (percent.HB) content. (B) UMAP of 20 clusters (0–19) from integrated asthma PBMC (*n* = 5) and AD CSF (*n* = 4) datasets, colored by cluster identity. (C) Clustifyr‐generated heatmap of transcriptional correlations between annotated cell types. (D) Annotated UMAP showing 11 cell types: B cells, CD14^+^ monocytes, CD16^+^ monocytes, CD4^+^ T cells, DCs, GZMK^+^ CD8^+^ T cells, macrophages, megakaryocytes, MHC‐II‐high CD14^+^ monocytes, NK cells, and pDCs. (E) Stacked bar plots of cell type proportions: (left) Asthma PBMCs show reduced MHC‐II‐high CD14^+^ monocytes versus normal PBMCs; (Right) AD CSF exhibits naive CD4^+^ T cell depletion versus normal CSF. Significance: **p < 0.001; **p < 0.01 (two‐tailed *t*‐test).

Integrated scRNA‐seq analysis of asthma PBMCs and AD CSF revealed distinct immune landscapes driven by tissue microenvironment and disease pathology. Quality control metrics (nFeature_RNA, nCount_RNA, percent.mt, percent.HB) confirmed data reliability, with 17,344 cells retained post‐QC (original: 19,115 cells; Figure 3A). Harmony‐based integration resolved batch effects, enabling cross‐tissue comparison. UMAP identified 20 transcriptionally distinct clusters (0–19; Figure 3B), which were annotated into 11 major cell types (Figure 3D): B cells, CD14^+^ monocytes, CD16^+^ monocytes, CD4^+^ T cells, dendritic cells (DCs), GZMK^+^ CD8^+^ T cells, macrophages, megakaryocytes, MHC‐II‐high CD14^+^ monocytes, natural killer (NK) cells, and plasmacytoid dendritic cells (pDCs). A clustifyr‐generated heatmap (Figure 3C) highlighted strong transcriptional correlations among T cell subsets (*r* = 0.72 for CD4^+^ vs. CD8^+^ T cells) and myeloid lineages (*r* = 0.65 for monocytes vs. macrophages). Tissue specificity was evident: megakaryocytes (PPBP^+^, Cluster 15) were exclusive to asthma PBMCs (2.1% of cells), while macrophages (CD68^+^, Cluster 18) dominated AD CSF (8.7% vs. 0% in PBMCs), consistent with monocyte differentiation in the central nervous system. Stacked bar plots (Figure 3E) revealed disease‐associated shifts: asthma PBMCs exhibited reduced MHC‐II‐high CD14^+^ monocytes (5.8% vs. 14.2% in normal PBMCs, *p* < 0.001) alongside expanded cytotoxic CD8^+^ T cells (12.7%) and plasma cells (6.3%), whereas AD CSF showed depletion of naive CD4^+^ T cells (9.3% vs. 19.4% in normal CSF, *p* < 0.01) and enrichment of neuroinflammatory macrophages (8.7%) and microglia (6.5%). These findings underscore tissue‐restricted immune dysfunction, with asthma characterized by adaptive immune activation and AD driven by myeloid‐mediated neuroinflammation.

### 3.7 Core Gene Expression and IL7R Subpopulation Communication Analysis

**FIGURE 4:**
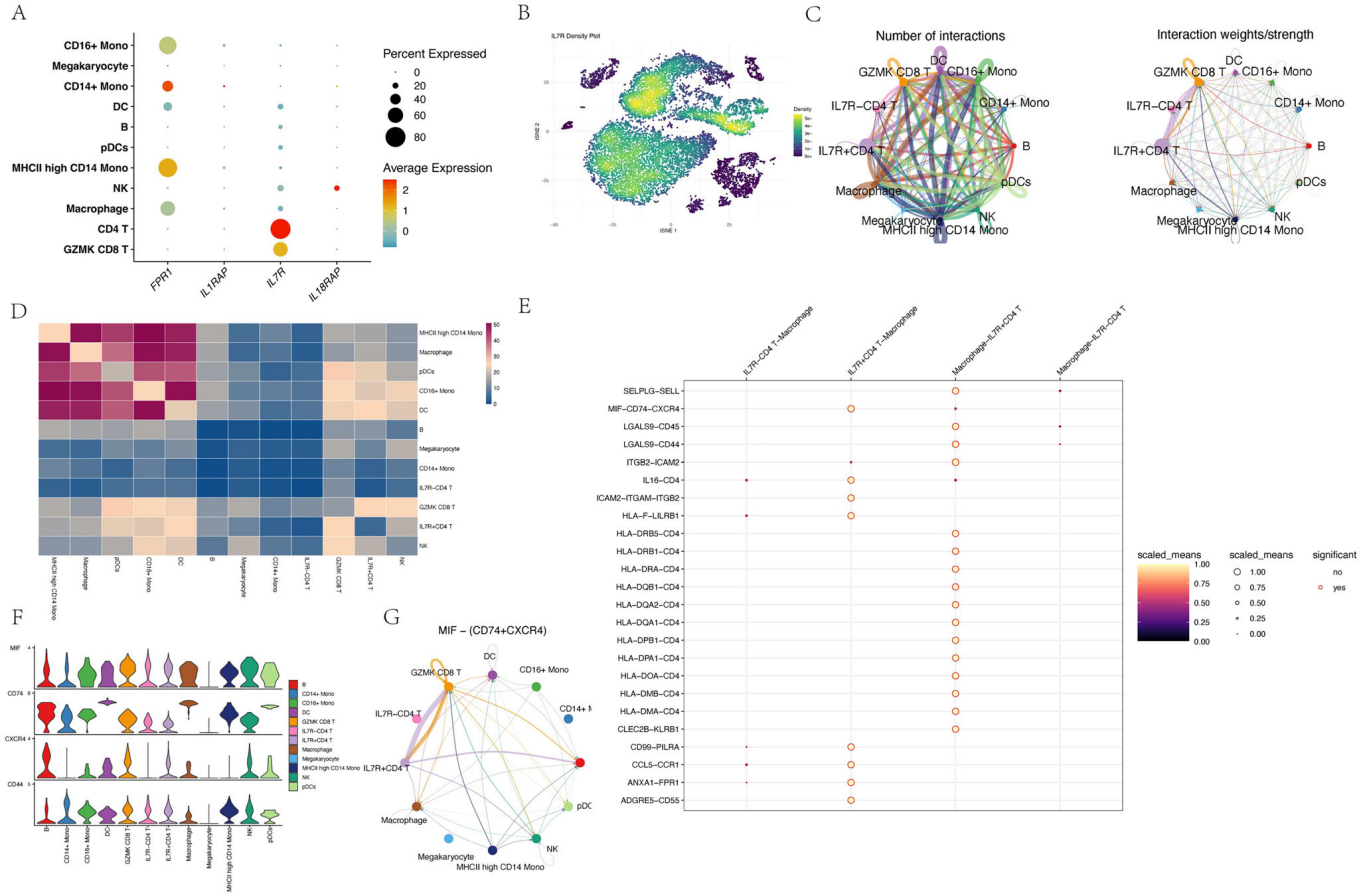
Core gene expression and IL7R subpopulation communication analysis. (A) Bubble plot: Expression of core genes across cell types, with bubble size reflecting the percentage of expressing cells and color indicating average expression. (B) Density plot: Distribution of IL7R expression in CD4 T cells, color‐graded by intensity. (C) Chord diagram: Number and weight of interactions between cell types, with arc thickness proportional to interaction strength. (D) Heatmap: Quantified interaction strengths between cell types (darker shades: stronger interactions). (E) Bubble Plot: Scaled expression of ligand–receptor pairs mediating intercellular communication. Bubble size reflects the inferred communication probability of the corresponding ligand–receptor pair in cell‐cell interactions. Bubble color intensity also represents this communication probability, with darker hues indicating higher probability values. Significant interactions (*p* < 0.05) are marked by a red halo. (F) Violin plots: Expression distributions of MIF, CD74, and CXCR4 across cell types. (G) Chord Diagram: Visualization of intercellular communication mediated by the MIF‐CD74‐CXCR4 signaling pathway. Nodes represent distinct cell populations. Arcs connecting nodes illustrate the predicted communication events between cell types. Arc thickness and color intensity are proportional to the inferred communication probability, indicating the strength of the interaction. Arrowheads on the arcs denote the direction of the signaling flow, originating from the signaling‐secreting cell type and pointing towards the target cell type. The diagram highlights the specific crosstalk between macrophages and IL7R^+^ CD4 T cells mediated by this pathway.

This study investigated the expression patterns of core genes and analyzed intercellular communication between IL7R‐defined CD4 T cell subpopulations. A bubble plot (Figure 4A) visualized the expression levels of genes across distinct cell types, with the average expression and percentage of cells expressing each gene quantified. Notably, IL7R exhibited high specificity and elevated expression in CD4 T cells, with an average expression value of approximately 2.5 and detection in over 80% of these cells. The distribution of IL7R expression within CD4 T cells was further validated by a density plot (Figure 4B), which confirmed its widespread presence. Based on this specificity, CD4 T cells were computationally stratified into IL7R^+^ and IL7R^−^ subpopulations using algorithms simulating knockdown and overexpression scenarios.

To elucidate the role of IL7R in cellular crosstalk, CellChat was employed to map communication networks among identified cell types. A chord diagram (Figure 4C) depicted the number and weighted strength of interactions, revealing macrophages as central communicators with both IL7R^+^ and IL7R^−^ CD4 T cells. The interaction strength between macrophages and IL7R^+^ CD4 T cells was particularly pronounced, as demonstrated by the thickness of connecting arcs. A heatmap (Figure 4D) further quantified these interactions, highlighting macrophages as dominant interactors with IL7R^+^ CD4 T cells (interaction strength: 0.82 vs. 0.41 for IL7R^−^ CD4 T cells). This observation prompted a focused analysis of macrophage‐driven communication.

A ligand–receptor pair bubble plot (Figure 4E) identified MIF‐CD74‐CXCR4 as a key pathway, with macrophages showing high expression of MIF (average expression: 3.1), while CD74 and CXCR4 were predominantly expressed in IL7R^+^ CD4 T cells (average expression: 2.8 and 2.6, respectively). Violin plots (Figure 4F) confirmed these expression patterns, with MIF localized to macrophages (detected in 92% of cells) and CD74/CXCR4 enriched in IL7R^+^ CD4 T cells (detected in 85% and 78% of cells, respectively).

The significance of the MIF‐CD74‐CXCR4 pathway was validated by a targeted chord diagram (Figure 4G). Interaction probabilities and statistical significance were calculated (*net_lr_1* data): macrophage‐to‐IL7R^+^ CD4 T cell interactions via this pathway showed a probability of *0.000478* (*p* = 0), indicating robust communication. In contrast, IL7R^+^ CD4 T cell self‐interactions exhibited negligible significance (probability: *0.00590*, *p* = 0.99). These results underscore the critical role of the MIF‐CD74‐CXCR4 axis in mediating macrophage‐CD4 T cell crosstalk, particularly within the IL7R^+^ subpopulation.

## 4 Discussion

As far as we know, no prospective studies have investigated the relationship of moderate to severe asthma with incident AD. In our study, two‐sample MR and MVMR analyses were utilized to evaluate the connection between moderate to severe asthma and AD, showing that moderate to severe asthma significantly increased the risk of AD (no reverse causality). Asthma and AD may be comorbid due to immunologic dysregulation and disturbance related to asthma, according to the neurobiological perspective (Ferretti and Cuello 2011; Griffin; Griffin and Barger 2010; Meraz‐Ríos et al. 2013; Michaud et al. 2013). Moderate to severe asthma is characterized by airway inflammation, which can have effects that can extend beyond the airways and impact brain health. For example, population‐based studies highlight the possible significance and influence of asthma on brain health, and these studies, while limited, have discovered an increased risk of dementia in individuals with asthma (Rusanen et al. 2013; M.‐H. Chen et al. 2014; Lutsey et al. 2019) and a greater risk in asthma patients with frequent or severe exacerbations (Peng et al. 2015). The aforementioned findings establish that eosinophil counts partially mediate the association between moderate to severe asthma and AD. Similar to the previous study by Zhang et al. (2022), they find that the eosinophil‐to‐lymphocyte ratio is significantly related to Aβ‐related biomarkers and eosinophils change dynamically along with disease progression in preclinical AD, indicating that peripheral eosinophils may contribute to the advancement of AD pathology.

The role of AD's pathophysiology is significant due to the release of proinflammatory cytokines during asthma's allergic responses. SMR results suggested that therapies targeting FPR1, IL1RAP, IL7R, and IL18RAP inflammatory variables may be able to prevent an increase in AD in moderate to severe asthma. IL‐1 is a contributing factor to the neurodegenerative process in AD, including activated dystrophic neurite formation in amyloid‐β peptide diffuse deposits (Griffin et al. 1995) and induction of astrocyte activation (Mrak and Griffin 2001). It is still noteworthy that FPR1 has negative effects on inflammatory diseases, which means that it may be considered as a therapeutic approach (Lammers et al. 2015). In addition, FPR1 can improve brain inflammation after intracerebral hemorrhage and neurological outcomes (Li et al. 2021). Passtoors et al. (2015) reveal that IL7R may impact the health status and rate of aging in older adults through examining the gene expression in blood. The variants of IL18 3′UTR can confer a survival advantage for motor neurons and protect against amyotrophic lateral sclerosis by reducing neuroinflammation (Eitan et al. 2022). Taken together, targeting these potential therapeutic targets may help delay AD progression.

Nonetheless, LDSC revealed no positive genetic correlation between the two diseases. We speculated that this result may be attributed to crosstalk between the different biological pathways in AD and moderate to severe asthma. Due to the subtle onset of AD, pinpointing the exact beginning of the condition is often challenging. Biomarkers in cerebrospinal fluid are being used more frequently to assist in diagnosing AD. Our hypothesis is that asthma‐related inflammation leads to central nervous system inflammation through PBMCs to CSF, which makes people more vulnerable to cognitive deterioration. It is worth noting that the IL7R may potentially play an important role in AD (according to the results of SMR and scRNA‐seq analysis). The potential role of IL7R in AD is supported by emerging evidence. For instance, Young et al. (2023) demonstrated via RT‐qPCR analysis that effector memory CD8^+^ T cells with low IL‐7Rα expression in the peripheral blood of AD patients exhibit distinct gene expression profiles compared to cognitively normal individuals, which negatively correlate with AD pathological markers (e.g., Aβ and tau) or cognitive scores. Furthermore, another study suggested that IL7R signaling may exacerbate neuroinflammation by modulating T cell infiltration and microglial activation (Dai and Shen 2021).

We found a critical role of the MIF‐CD74‐CXCR4 axis in mediating macrophage‐CD4 T cell crosstalk, particularly within the IL7R^+^ subpopulation. MIF is significantly increased in the serum of asthma patients and promotes Th2 cell differentiation and eosinophil infiltration by activating CD74 receptors (Bozza et al. 2020). Meanwhile, MIF levels are increased in the cerebrospinal fluid of AD patients and are positively correlated with microglial activation and Aβ deposition (Nasiri et al. 2020). Peripheral immune cells (such as CD4^+^ T cells) in asthma highly express CXCR4, and its ligand CXCL12 is upregulated in the perivascular areas of the brain of AD patients, which may attract peripheral immune cells to cross the blood–brain barrier and enter the brain parenchyma (Korte et al. 2024). Infiltrating CD4^+^ T cells (such as Th1/Th17) secrete IFN‐γ and IL‐17, further activating microglia, forming an inflammatory positive feedback loop, and exacerbating Aβ deposition and neuronal damage. According to the AD model, inhibiting CXCR4 can reduce the infiltration of peripheral immune cells in the brain and improve cerebral blood flow and cognitive function (Korte et al. 2024).

The effect size of moderate‐to‐severe asthma in AD (OR_IVW _= 1.01, *p *= 3.85 × 10^−3^) is statistically significant but biologically weak. The statistically insignificant results may be due to insufficient statistical power, indicating that the current sample size is not enough to detect small effects. However, the effect estimates of other MR analysis methods are in the same direction (such as OR slightly > 1), which may indicate a potential risk trend and requires larger sample verification. Although the causal effect size of moderate to severe asthma and AD (OR = 1.01–1.04) is lower than that of known genetic risk factors (such as the APOE ε4 allele, OR = 3–15) (Zheng and Wang 2025; Kim et al. 2025; Wingo et al. 2021), it has unique public health significance as a modifiable exposure factor. For example, APOE ε4 cannot be intervened as a genetic marker, while the inflammatory pathways of asthma (such as IL‐7R signaling) can be targeted through drugs (such as anti‐IL‐7R antibodies) or lifestyle adjustments. If future studies confirm that asthma management can reduce the risk of AD, even a small effect size may translate into a significant reduction in disease burden due to the high prevalence of asthma.

Our findings broaden our knowledge of the processes that asthma and AD share, indicating that immune cells from asthma PBMSs may interact with CSF to influence the central nervous system through inflammatory factors. Dysregulation of the inflammatory pathway might impact the central nervous system and play a role in the pathophysiology of neurodegenerative disorders. Thus, to better identify the inflammatory pathogenic pathways correlated to altered brain health in asthma, a thorough investigation of the inflammatory pathways and their interactions is required. Treatments aimed at inflammatory factors may potentially prevent an increase in AD among patients with moderate to severe asthma. To achieve further therapeutic advancements, it is crucial to understand the inflammatory pathways linking moderate to severe asthma and AD, which could mitigate or delay the onset of neurodegeneration and dementia. Future research will emphasize the importance of addressing the risk of neurodegeneration and cognitive impairment in the large population of asthma patients.

## 5 Conclusion

This study has several limitations. First, we selected GWAS data with sufficient statistical power to investigate the causality between moderate to severe asthma and AD. However, residual confounding factors and horizontal pleiotropy cannot be completely eliminated. Second, the lack of comprehensive study‐level data precluded any further subgroup analyses. Third, the MR analysis causal estimate reflected the impact of drug targets over a person's lifetime; thus, the short‐term effectiveness and adverse effects could not be assessed. Fourth, restricted cross‐ancestry generalizability due to Eurocentric genetic instruments, potentially biasing causal estimates in underrepresented populations. Fifth, scRNA‐seq data are vulnerable to sample‐specific technical artifacts (e.g., dropout‐driven false negatives) and biological heterogeneity (e.g., inter‐individual immune variation), which may confound cell‐type‐resolved causal inference. Future studies should prioritize diverse cohorts and multimodal single‐cell assays.

## Author Contributions

**Jing Yang**: formal analysis, writing–original draft. **Zixing Liu**: validation, writing–original draft, data curation. **Xiaofang Li**: writing–original draft, formal analysis. **Yichong Qiu**: data curation, validation. **Qiong Liu**: funding acquisition, supervision. **Xiufang Huang**: writing–review and editing, funding acquisition, supervision. **Leshen Lian**: conceptualization, investigation, funding acquisition, writing–review and editing.

## Conflicts of Interest

The authors declare no conflicts of interest.

## Ethics Statement

The authors have nothing to report.

## Peer Review

The peer review history for this article is available at https://publons.com/publon/10.1002/brb3.70809.

## Supporting information




**Supplementary Materials**: brb370809‐sup‐0001‐Tables.docx

## Data Availability

All data generated or analyzed during this study are included in this article.
