# Supplementary material for: IL7R+ T Cell‐Macrophage Crosstalk Links Asthma to Alzheimer's Pathogenesis: Integrating Mendelian Randomization and CellChat Analysis
Source: Brain Behav. 2025 Sep 15;15(9):e70809. doi: 10.1002/brb3.70809 (PMC12434707; doi:10.1002/brb3.70809)
Supplement: Supplementary file 1 — Supplementary Materials: brb370809‐sup‐0001‐Tables.docx [file BRB3-15-e70809-s001.docx]

Contents:

Supplementary Table 1. Information of eQTL and GWAS summary data.

Supplementary Table 2. Overview of cohorts for meta-analysis of Alzheimer’s disease.

Supplementary Table 3. Baseline characteristics of stage 1 and stage 2 cohorts.

Supplementary Table 4. SNPs of exposure and mediators used in MR analyses.

Supplementary Table 5. Association between moderate to severe asthma and AD adjusted for BMI, diabetes, hypertension, cigarettes smoked per day or total cholesterol in multivariable MR analysis.

Supplementary Table 6. Reverse two-step MR association between AD and moderate to severe asthma.

Supplementary Table 7. Information of genetic instrumental variants associated with inflammatory within 100 kb windows from gene FPR1, IL1RAP, IL7R or IL18RAP.

Supplementary Table 8. SMR association between expression of gene FPR1, IL1RAP, IL7R, or IL18RAP and AD outcomes.

Supplementary Table 9. Association between eQTL top SNP of FPR1 with expression of other nearby genes (*P*<0.05).

Supplementary Table 10. IVW-MR association between inflammatory factory mediated by gene FPR1, IL1RAP, IL7R, or IL18RAP and repeat AD GWAS.

Supplementary Table 11. IVW-MR association between inflammatory factory mediated by gene FPR1, IL1RAP, IL7R, or IL18RAP and asthma.

Supplementary Table 12. the LD Score regression intercept, heritability and genetic correlation.

Supplementary Table 1. Information of eQTL and GWAS summary data.

| **Characteristic** | **Resource** | **Sample size** | **Population ancestry** | **Reference** | **Data download** |
| --- | --- | --- | --- | --- | --- |
| **eQTL data** |  |  |  |  |  |
| eQTL for FPR1, IL1RAP,IL7R, IL18RAP | eQTLGen Consortium | Whole blood: 31684 | Predominantly European | [Võsa, U., Claringbould, A.,Franke, L, et al(2018): Unraveling the polygenic architecture of complex traits using blood eQTL meta-analysis. https://www.biorxiv.org/content/10.1101/447367v1](https://www.biorxiv.org/content/10.1101/447367v1) | <https://www.eqtlgen.org/cis-eqtls.html> |
| **GWAS summary data** |  |  |  |  |  |
| Alzheimer's disease | Nature Genetics | Number of cases:71,880 Number of controls: 383,378 | European | Genome-wide meta-analysis identifies new loci and functional pathways influencing Alzheimer's disease risk(2019). https://pubmed.ncbi.nlm.nih.gov/30617256/ | https://www.ebi.ac.uk/gwas/home |
| Moderate to severe asthma | The Lancet. Respiratory medicine | Number of cases:10,549 Number of controls: 47,146 | European | Moderate-to-severe asthma in individuals of European ancestry: a genome-wide association study(2018). https://pubmed.ncbi.nlm.nih.gov/30552067/ | https://www.ebi.ac.uk/gwas/home |
| Eosinophil counts | Nature Genetics | Number of cases and controls:442,919 | Predominantly European | A cross-population atlas of genetic associations for 220 human phenotypes(2021). https://pubmed.ncbi.nlm.nih.gov/34594039/ | https://www.ebi.ac.uk/gwas/home |
| Interleukin-1 Receptor accessory protein, Interleukin-7 receptor subunit alpha, Interleukin-18 receptor 1 | Nature | Number of cases and controls:3301 | European | [Genomic atlas of the human plasma proteome (2018). https://pubmed.ncbi.nlm.nih.gov/29875488/](https://pubmed.ncbi.nlm.nih.gov/24097068/) | https://gwas.mrcieu.ac.uk/ |
| **Repeat verification GWAS summary data** |  |  |  |  |  |
| eosinophil cell count | Blood Cell Consortium | Number of cases and controls: 563,946 | European | [The Polygenic and Monogenic Basis of Blood Traits and Diseases (2020). https://pubmed.ncbi.nlm.nih.gov/32888494/](https://pubmed.ncbi.nlm.nih.gov/26343387/) | https://gwas.mrcieu.ac.uk/ |
| asthma | MRC-IEU | Number of cases:18,988 Number of controls: 444,022 | European | [/](https://pubmed.ncbi.nlm.nih.gov/26343387/) | https://gwas.mrcieu.ac.uk/ |
| Alzheimer's disease or family history of Alzheimer's disease | Nature Genetics | Number of cases and controls: 472,868 | European | [Genome-wide meta-analysis, fine-mapping and integrative prioritization implicate new Alzheimer's disease risk genes(2021). https://pubmed.ncbi.nlm.nih.gov/33589840/](https://pubmed.ncbi.nlm.nih.gov/26343387/) | https://gwas.mrcieu.ac.uk/ |
| Diabetes | Nature Genetics | Number of cases:21,969 Number of controls: 433,048 | European | [A generalized linear mixed model association tool for biobank-scale data (2021). https://pubmed.ncbi.nlm.nih.gov/34737426/](https://pubmed.ncbi.nlm.nih.gov/26343387/) | https://www.ebi.ac.uk/gwas/home |
| Body mass index | Nature Communications | Number of cases and controls: 694,649 | European | [Pleiotropic genetic architecture and novel loci for C-reactive protein levels (2022). https://pubmed.ncbi.nlm.nih.gov/36376304/](https://pubmed.ncbi.nlm.nih.gov/26343387/) | https://www.ebi.ac.uk/gwas/home |
| Cigarettes smoked per day | Nature Communications | Number of cases and controls: 337,334 | European | [Pleiotropic genetic architecture and novel loci for C-reactive protein levels (2022). https://pubmed.ncbi.nlm.nih.gov/36376304/](https://pubmed.ncbi.nlm.nih.gov/26343387/) | https://www.ebi.ac.uk/gwas/home |
| Total cholesterol | Nature Communications | Number of cases and controls: 21,491 | European | [Genome-wide study for circulating metabolites identifies 62 loci and reveals novel systemic effects of LPA (2016). https://pubmed.ncbi.nlm.nih.gov/27005778/](https://pubmed.ncbi.nlm.nih.gov/26343387/) | https://www.ebi.ac.uk/gwas/home |
| Hypertension | Nature Aging | Number of cases:129,909 Number of controls: 354,689 | European | [Common genetic associations between age-related diseases (2021). https://pubmed.ncbi.nlm.nih.gov/33959723/](https://pubmed.ncbi.nlm.nih.gov/26343387/) | https://www.ebi.ac.uk/gwas/home |

Supplementary Table 2. Overview of cohorts for meta-analysis of Alzheimer’s disease.

| **Cohort** | **Sub-cohort** | **Geographic origin** | **N** | **(proxy) cases** | | | **(proxy) controls** | | | **Phenotype** | **Data Type** | **Imputation Reference** |
| --- | --- | --- | --- | --- | --- | --- | --- | --- | --- | --- | --- | --- |
|  |  |  |  | **N** | **Age of onset** | **% female** | **N** | **Age last contact** | **% female** |  |  |  |
| 1. PGC-ALZ* | a. DemGene | Norway | 9703 | 2066 | 77.0 | 62.9 | 7637 | 58.8 | 48.0 | AD | GWAS | 1000G Phase 3 |
|  | b. TwinGene | Sweden | 6647 | 322 | 79.6 | 52.5 | 6325 | 64.2 | 52.4 | AD | GWAS | 1000G Phase 3 |
|  | c. STSA | Sweden | 1127 | 348 | 80.5 | 66.4 | 779 | 75.5 | 51.1 | AD | GWAS | 1000G Phase 3 |
| 2. IGAP |  | Europe/US | 54162 | 17008 | 74.2 | 61.3 | 37154 | 69.8 | 57.3 | AD | GWAS | 1000G Phase 1 |
| 3. ADSP |  | Europe/US | 7506 | 4343 | 74.1 | 55.0 | 3163 | 86.5 | 59.5 | AD | WES | 1000G Phase 3 |
| 4. UKB |  | UK | 376113 | 47793 | 59.1 | 56.5 | 328320 | 56.4 | 53.6 | by proxy AD | GWAS | HRC |
| Sum |  |  |  | 71880 |  |  | 383,378 |  |  |  |  |  |

Note: Date cited from Genome-wide meta-analysis identifies new loci and functional pathways influencing Alzheimer’s disease risk (PMID: 30617256). * The PGC-ALZ summary statistic have been meta-analyzed with the other 3 main cohorts, but PGC-ALZ sub-cohort specific demographics are also displayed. The 'age of onset' and 'age of last contact' of the UKB samples, refers to the age of the UKB participants (so not of the parents). For the controls of the DemGene cohort, mean age was based on 2382 individuals for which this information was available. Proxy cases and controls only refers to the UKB cohort, all other cohorts are clinical case-control cohorts. AD = Alzheimer's disease; GWAS = genome-wide genotype data; WES = whole exome sequencing data; 1000G = Thousand Genomes; HRC = Haplotype Reference Consortium.

Supplementary Table 3. Baseline characteristics of stage 1 and stage 2 cohorts.

|  |  | **Stage 1 cohort** | | **Stage 2 cohort** | | |
| --- | --- | --- | --- | --- | --- | --- |
|  |  | **Cases (n=5135)** | **Controls (n=25 675)** | **Cases (n=5414)** | **Controls (n=21 471)** | **Controls for sensitivity analyses*(n=27 082)** |
| Age, years |  | 55 (12) | 56 (8) | 58 (8) | 58 (8) | 58 (8) |
| Sex |  |  |  |  |  |  |
|  | Female | 3170 (61·7%) | 14 626 (57·0%) | 3354 (62·0%) | 13 135 (61·2%) | 16 816 (62·1%) |
|  | Male | 1965 (38·3%) | 11 049 (43·0%) | 2060 (38·0%) | 8336 (38·8%) | 10 266 (37·9%) |
|  | FEV1, % predicted | 72·4% (21·4) | 91·8% (17·4) | 84·5% (17·2) | 93·7% (14·1) | 93·9% (13·9) |
|  | FEV1/FVC | 0·67 (0·12) | 0·76 (0·06) | 0·73 (0·09) | 0·77 (0·06) | 0·77 (0·06) |
| Smoking status |  |  |  |  |  |  |
|  | Ever smoker | 2265 (44·1%) | 11 913 (46·4%) | 2509 (46·3%) | 9479 (44·2%) | 11 707 (43·2%) |
|  | Never smoker | 2647 (51·6%) | 13 487 (52·5%) | 2787 (51·5%) | 11 621 (54·1%) | 14 918 (55·1%) |
|  | Unknown | 223 (4·3%) | 275 (1·1%) | 118 (2·2%) | 371 (1·7%) | 457 (1·7%) |
| Rhinitis or eczema status |  |  |  |  |  |  |
|  | Yes | 1897 (36·9%) | 8† | 2556 (47·2%) | 0 | 5541 (20·5%) |
|  | No | 2062 (40·2%) | 25 667† | 2858 (52·8%) | 21 471 (100%) | 21 541 (79·5%) |
|  | Unknown | 1176 (22·9%) | 0† | 0 | 0 | 0 |
|  | Oral corticosteroid use (prednisolone) | 222/3710 (6·0%) | NA | 162/5414 (3·0%) | NA | NA |

Note: Data cited from Moderate-to-severe asthma in individuals of European ancestry: a genome-wide association study (PMID: 30552067). Data are mean (SD) or n (%), unless otherwise stated. FEV1=forced expiratory volume in 1 s. FVC=forced vital capacity. NA=not applicable. U-BIOPRED=Unbiased BIOmarkers in PREDiction of respiratory disease outcomes. *Including all controls with rhinitis, eczema, and allergy. †Patients in the U-BIOPRED cohort were not screened for rhinitis or eczema before sample selection but were subsequently found to comprise eight patients with rhinitis, eczema, or allergy.

Supplementary Table 4. SNPs of exposure and mediators used in MR analyses.

| **Exposure** | **SNP** | **Effect allele** | **Other allele** | **Effect allele frequency** | **beta** | **se** | ***p*-value** | **F-statistics** |
| --- | --- | --- | --- | --- | --- | --- | --- | --- |
| Moderate to severe asthma | rs7523907 | T | C | 0.541 | 0.135 | 0.0224 | 1.64E-09 | 17.57873967 |
|  | rs12479210 | T | C | 0.387 | 0.183 | 0.0225 | 4.77E-16 | 31.07356613 |
|  | rs34290285 | A | G | 0.257 | -0.193 | 0.0251 | 1.41E-14 | 27.77284766 |
|  | rs1438673 | T | C | 0.492 | -0.169 | 0.0221 | 2.11E-14 | 27.87460465 |
|  | rs3749833 | C | T | 0.261 | 0.161 | 0.0249 | 1.14E-10 | 19.43849412 |
|  | rs9273410 | A | C | 0.553 | 0.23 | 0.0225 | 1.07E-24 | 51.68433835 |
|  | rs13274782 | C | A | 0.623 | -0.132 | 0.0228 | 7.29E-09 | 15.88210064 |
|  | rs144829310 | T | G | 0.164 | 0.207 | 0.0297 | 3.68E-12 | 22.88153952 |
|  | rs61840192 | A | G | 0.427 | -0.167 | 0.0224 | 8.42E-14 | 26.62837316 |
|  | rs11603634 | G | A | 0.504 | 0.124 | 0.0223 | 2.30E-08 | 14.90968869 |
|  | rs7936312 | T | G | 0.474 | 0.134 | 0.022 | 1.09E-09 | 17.3876624 |
|  | rs4622308 | T | C | 0.53 | -0.127 | 0.0225 | 1.69E-08 | 15.59001296 |
|  | rs703816 | C | T | 0.434 | 0.151 | 0.0222 | 1.18E-11 | 21.80287999 |
|  | rs72743461 | A | C | 0.236 | 0.167 | 0.0258 | 1.03E-10 | 19.55192687 |
|  | rs7203459 | C | T | 0.246 | -0.206 | 0.0255 | 7.83E-16 | 30.78176844 |
|  | rs112502960 | A | G | 0.359 | 0.134 | 0.023 | 6.05E-09 | 16.03715999 |
| Sum |  |  |  |  |  |  |  | **376.8757032** |
| Eosinophil counts | rs10057057 | A | G | 0.2147938 | -0.0141 | 0.002 | 9.56E-13 | 0.065797158 |
|  | rs10062687 | G | T | 0.1870774 | 0.0157 | 0.0021 | 4.35E-14 | 0.07355916 |
|  | rs10068965 | A | G | 0.5194524 | 0.0093 | 0.0016 | 1.61E-08 | 0.042364409 |
|  | rs10123514 | C | T | 0.3825604 | 0.01 | 0.0017 | 3.08E-09 | 0.046349957 |
|  | rs10127666 | A | G | 0.0689378 | -0.0225 | 0.0035 | 8.80E-11 | 0.063762262 |
|  | rs1012919 | G | T | 0.6514738 | 0.0143 | 0.0017 | 8.96E-17 | 0.091112746 |
|  | rs1019527 | G | A | 0.2764676 | 0.012 | 0.0019 | 5.16E-10 | 0.056522853 |
|  | rs10261508 | C | T | 0.2464392 | -0.0386 | 0.0019 | 3.89E-92 | 0.543222043 |
|  | rs10411344 | C | A | 0.1295685 | 0.0178 | 0.0024 | 7.54E-14 | 0.070119589 |
|  | rs1041973 | A | C | 0.214752 | -0.0355 | 0.002 | 3.19E-72 | 0.417176337 |
|  | rs10445361 | G | T | 0.3112762 | 0.0226 | 0.002 | 1.25E-29 | 0.21490047 |
|  | rs1047891 | A | C | 0.2814723 | 0.0141 | 0.0018 | 9.06E-15 | 0.078901752 |
|  | rs10488849 | T | C | 0.5188253 | -0.0094 | 0.0017 | 1.08E-08 | 0.043284573 |
|  | rs10508372 | A | G | 0.1596747 | -0.0285 | 0.003 | 7.09E-22 | 0.213895814 |
|  | rs1057258 | T | C | 0.2116207 | -0.0201 | 0.0021 | 1.96E-22 | 0.132275178 |
|  | rs10764617 | C | A | 0.3410239 | -0.0209 | 0.0017 | 1.84E-33 | 0.192648968 |
|  | rs10786335 | T | C | 0.6073725 | 0.0106 | 0.0017 | 2.60E-10 | 0.052578134 |
|  | rs10808714 | C | T | 0.4640443 | 0.0124 | 0.0017 | 5.48E-14 | 0.075041112 |
|  | rs10887908 | A | G | 0.3050704 | 0.0124 | 0.0021 | 1.47E-09 | 0.063965668 |
|  | rs10900522 | C | T | 0.2626895 | 0.0105 | 0.0019 | 2.33E-08 | 0.041901017 |
|  | rs10902457 | T | C | 0.5933109 | 0.0099 | 0.0018 | 1.65E-08 | 0.04640557 |
|  | rs10905467 | T | C | 0.5184164 | -0.0139 | 0.0017 | 1.42E-16 | 0.094657769 |
|  | rs10950805 | G | C | 0.3639089 | -0.0113 | 0.0017 | 3.59E-11 | 0.058000123 |
|  | rs10956403 | G | A | 0.307647 | -0.0223 | 0.0018 | 1.17E-36 | 0.207881721 |
|  | rs10956485 | C | T | 0.3045355 | -0.0246 | 0.0018 | 1.07E-41 | 0.251552567 |
|  | rs10975479 | G | A | 0.1305399 | 0.0627 | 0.0024 | 3.73E-155 | 0.876294782 |
|  | rs11010129 | C | T | 0.3238642 | 0.0113 | 0.0017 | 9.67E-11 | 0.054867144 |
|  | rs11126315 | C | T | 0.3858941 | -0.0102 | 0.0019 | 4.58E-08 | 0.048380184 |
|  | rs111796602 | C | T | 0.0833943 | 0.0269 | 0.0029 | 6.58E-21 | 0.108543955 |
|  | rs11185551 | C | G | 0.1646756 | -0.0168 | 0.0022 | 1.18E-14 | 0.076185294 |
|  | rs111862617 | T | A | 0.0189709 | -0.0361 | 0.0059 | 7.40E-10 | 0.047592615 |
|  | rs112173287 | C | T | 0.2259 | -0.015 | 0.0021 | 1.28E-12 | 0.077208361 |
|  | rs112425105 | T | C | 0.0536106 | 0.0268 | 0.0039 | 5.97E-12 | 0.071508244 |
|  | rs11243920 | T | C | 0.0231605 | 0.0406 | 0.0059 | 6.67E-12 | 0.073179585 |
|  | rs11253530 | C | T | 0.216115 | 0.0231 | 0.0021 | 3.07E-27 | 0.177408364 |
|  | rs112691446 | A | G | 0.1706518 | 0.0456 | 0.0021 | 1.36E-101 | 0.57778656 |
|  | rs112708167 | T | C | 0.0293452 | -0.0331 | 0.0053 | 5.32E-10 | 0.061237763 |
|  | rs113315602 | C | A | 0.078427 | 0.0256 | 0.0031 | 2.11E-16 | 0.09295034 |
|  | rs113429444 | T | C | 0.0823469 | -0.019 | 0.0032 | 2.83E-09 | 0.053529234 |
|  | rs113473633 | G | A | 0.0262107 | -0.0338 | 0.0057 | 3.62E-09 | 0.057218529 |
|  | rs113528897 | C | T | 0.294498 | 0.011 | 0.0019 | 1.24E-08 | 0.049331253 |
|  | rs113542380 | A | G | 0.0750823 | -0.021 | 0.0034 | 4.80E-10 | 0.060095256 |
|  | rs113844630 | C | T | 0.1161691 | -0.0177 | 0.0026 | 2.54E-11 | 0.063120248 |
|  | rs114438813 | C | T | 0.0139899 | -0.0432 | 0.0075 | 9.06E-09 | 0.050514978 |
|  | rs114561028 | G | A | 0.0158642 | 0.0477 | 0.0071 | 1.64E-11 | 0.069706802 |
|  | rs114741563 | C | T | 0.0072501 | 0.078 | 0.0104 | 4.84E-14 | 0.085930475 |
|  | rs114925538 | T | C | 0.0461458 | -0.0225 | 0.0039 | 7.63E-09 | 0.043725337 |
|  | rs11538263 | A | G | 0.0362456 | 0.0275 | 0.0042 | 7.28E-11 | 0.051837598 |
|  | rs11540540 | C | A | 0.2776593 | -0.0114 | 0.0018 | 4.72E-10 | 0.051147092 |
|  | rs115478735 | T | A | 0.1557984 | -0.0184 | 0.0022 | 1.17E-16 | 0.087381177 |
|  | rs11555542 | C | T | 0.063872 | 0.0399 | 0.0036 | 1.40E-28 | 0.186813635 |
|  | rs115820364 | T | C | 0.0585385 | 0.0294 | 0.0038 | 4.15E-15 | 0.093479102 |
|  | rs11588625 | C | T | 0.1066662 | -0.0177 | 0.0026 | 5.20E-12 | 0.058579751 |
|  | rs115976537 | A | G | 0.029747 | 0.031 | 0.0055 | 1.43E-08 | 0.054426447 |
|  | rs11616188 | A | G | 0.3383762 | -0.0116 | 0.0018 | 6.55E-11 | 0.059113604 |
|  | rs11622216 | C | A | 0.3209709 | -0.0116 | 0.0018 | 6.49E-11 | 0.057547951 |
|  | rs11624726 | T | C | 0.365058 | -0.0101 | 0.0017 | 5.48E-09 | 0.046397387 |
|  | rs11643541 | C | T | 0.2341046 | 0.0215 | 0.002 | 4.39E-27 | 0.162653206 |
|  | rs11649485 | G | A | 0.1807964 | 0.023 | 0.0021 | 1.78E-27 | 0.15375871 |
|  | rs116894976 | T | C | 0.0702152 | 0.0305 | 0.0035 | 9.39E-19 | 0.119179169 |
|  | rs11693463 | G | A | 0.0877782 | -0.0175 | 0.003 | 8.35E-09 | 0.048119229 |
|  | rs11700925 | T | C | 0.2106755 | 0.0111 | 0.002 | 2.16E-08 | 0.040203855 |
|  | rs11702689 | T | C | 0.0947367 | -0.0209 | 0.0027 | 1.49E-14 | 0.073511079 |
|  | rs117068593 | T | C | 0.189781 | -0.0172 | 0.0022 | 2.21E-14 | 0.089265964 |
|  | rs117315456 | A | C | 0.018844 | -0.035 | 0.0058 | 1.90E-09 | 0.044442805 |
|  | rs117641185 | A | C | 0.0207748 | -0.0401 | 0.0062 | 1.07E-10 | 0.064190526 |
|  | rs117683492 | A | G | 0.0184948 | 0.0415 | 0.0065 | 1.86E-10 | 0.061347933 |
|  | rs117710327 | A | C | 0.0598315 | -0.0257 | 0.0035 | 1.14E-13 | 0.072906879 |
|  | rs11810687 | C | T | 0.3932431 | 0.0128 | 0.0018 | 5.12E-13 | 0.076712138 |
|  | rs118148121 | A | T | 0.0515486 | -0.0356 | 0.0041 | 1.36E-18 | 0.121596232 |
|  | rs118159012 | C | G | 0.0214272 | -0.0432 | 0.0056 | 2.01E-14 | 0.07678833 |
|  | rs11975761 | A | G | 0.7675916 | -0.0125 | 0.0019 | 7.38E-11 | 0.054696635 |
|  | rs11999525 | A | G | 0.2311525 | 0.0107 | 0.0019 | 2.71E-08 | 0.039926231 |
|  | rs12122584 | T | A | 0.097161 | -0.0193 | 0.0029 | 5.41E-11 | 0.064117949 |
|  | rs1214596 | A | T | 0.3277793 | -0.0116 | 0.0018 | 5.17E-11 | 0.058179429 |
|  | rs12208103 | T | C | 0.3457926 | -0.0211 | 0.0017 | 3.19E-34 | 0.197659588 |
|  | rs12338765 | C | A | 0.4637942 | -0.0136 | 0.0017 | 1.69E-16 | 0.090262828 |
|  | rs12359396 | T | C | 0.0671952 | 0.0243 | 0.0035 | 5.26E-12 | 0.072628665 |
|  | rs1236176 | A | T | 0.3316149 | 0.0134 | 0.0018 | 1.48E-13 | 0.078097887 |
|  | rs12420857 | T | G | 0.0657187 | 0.028 | 0.0034 | 1.53E-16 | 0.094462303 |
|  | rs12434881 | A | G | 0.4517718 | 0.0209 | 0.0016 | 5.32E-37 | 0.212325124 |
|  | rs12469015 | T | C | 0.3268754 | 0.0108 | 0.0018 | 1.06E-09 | 0.05035955 |
|  | rs12488267 | T | A | 0.3110034 | 0.0102 | 0.0018 | 1.21E-08 | 0.043745787 |
|  | rs12619285 | G | A | 0.3529777 | -0.0417 | 0.0018 | 2.19E-114 | 0.779862547 |
|  | rs12624544 | C | T | 0.2502351 | -0.0116 | 0.0019 | 8.29E-10 | 0.049538817 |
|  | rs12635478 | A | C | 0.3683251 | 0.01 | 0.0017 | 8.23E-09 | 0.045654067 |
|  | rs12637184 | A | G | 0.3284796 | 0.0151 | 0.0019 | 5.97E-16 | 0.098695994 |
|  | rs1264563 | T | C | 0.384482 | -0.0215 | 0.0017 | 2.15E-37 | 0.214695566 |
|  | rs12705849 | A | G | 0.406739 | -0.0143 | 0.0018 | 1.52E-15 | 0.096830214 |
|  | rs12713806 | G | T | 0.2673372 | -0.0107 | 0.0018 | 5.70E-09 | 0.044003269 |
|  | rs12718488 | G | T | 0.3577848 | 0.0125 | 0.0017 | 2.83E-13 | 0.070451139 |
|  | rs12738352 | C | T | 0.5773003 | -0.0102 | 0.0018 | 7.86E-09 | 0.049818479 |
|  | rs12799471 | A | G | 0.6802932 | -0.0108 | 0.0019 | 5.07E-09 | 0.049779654 |
|  | rs12820863 | T | C | 0.318263 | 0.0157 | 0.0018 | 2.01E-18 | 0.104950167 |
|  | rs12861824 | C | T | 0.272826 | -0.0119 | 0.0021 | 1.63E-08 | 0.055128575 |
|  | rs12882644 | A | G | 0.3375049 | -0.026 | 0.0018 | 1.23E-46 | 0.296671009 |
|  | rs12885373 | A | G | 0.1947492 | -0.0122 | 0.0021 | 2.66E-09 | 0.045801642 |
|  | rs12931183 | G | A | 0.6441992 | -0.0203 | 0.0019 | 1.05E-25 | 0.185368344 |
|  | rs12952003 | C | T | 0.6173906 | 0.0109 | 0.0017 | 1.56E-10 | 0.05507156 |
|  | rs12963021 | T | C | 0.6711196 | 0.0154 | 0.0018 | 5.43E-18 | 0.102720975 |
|  | rs12974418 | A | G | 0.372777 | -0.0117 | 0.002 | 2.23E-09 | 0.062806552 |
|  | rs12985107 | G | A | 0.3346247 | 0.0126 | 0.0017 | 5.05E-13 | 0.069363468 |
|  | rs13023118 | G | A | 0.095503 | 0.0372 | 0.0029 | 2.27E-37 | 0.234610821 |
|  | rs13048344 | C | A | 0.4017471 | 0.0143 | 0.0017 | 1.89E-17 | 0.096446547 |
|  | rs13088560 | T | G | 0.177557 | -0.0193 | 0.0024 | 6.49E-16 | 0.106743101 |
|  | rs13170453 | G | A | 0.2011852 | 0.0119 | 0.002 | 4.51E-09 | 0.044657056 |
|  | rs13213149 | C | T | 0.2170156 | -0.0352 | 0.0025 | 3.62E-46 | 0.413282104 |
|  | rs13219558 | C | T | 0.329539 | 0.0386 | 0.0019 | 1.67E-94 | 0.646361261 |
|  | rs13223928 | C | T | 0.4831684 | -0.0149 | 0.0017 | 2.10E-19 | 0.108793416 |
|  | rs13256023 | T | C | 0.1846164 | -0.0209 | 0.0021 | 2.32E-23 | 0.12903762 |
|  | rs1326125 | C | A | 0.3212545 | 0.0099 | 0.0017 | 1.66E-08 | 0.041935274 |
|  | rs13315649 | A | T | 0.276707 | 0.0251 | 0.002 | 7.14E-37 | 0.247471804 |
|  | rs13407652 | A | G | 0.3483219 | 0.0104 | 0.0017 | 2.92E-09 | 0.048176611 |
|  | rs13423778 | G | A | 0.223117 | 0.0133 | 0.0021 | 2.84E-10 | 0.060166191 |
|  | rs1343852 | G | C | 0.3264542 | 0.0159 | 0.0017 | 6.49E-20 | 0.109085312 |
|  | rs13447454 | A | G | 0.1174439 | 0.014 | 0.0025 | 1.34E-08 | 0.039863999 |
|  | rs1375493 | A | G | 0.5184949 | 0.012 | 0.0017 | 5.28E-13 | 0.07054617 |
|  | rs138642871 | A | G | 0.04833 | -0.0247 | 0.0042 | 3.61E-09 | 0.055062483 |
|  | rs140543080 | G | A | 0.0578413 | -0.04 | 0.0041 | 1.01E-22 | 0.171116473 |
|  | rs140656232 | T | G | 0.0190404 | 0.0392 | 0.0062 | 2.96E-10 | 0.056319411 |
|  | rs1420101 | T | C | 0.3903146 | 0.0498 | 0.0017 | 2.71E-194 | 1.159381958 |
|  | rs142487205 | T | C | 0.0298318 | 0.0302 | 0.0052 | 6.18E-09 | 0.05179617 |
|  | rs1434282 | T | C | 0.7429831 | -0.0231 | 0.0019 | 3.04E-35 | 0.199980422 |
|  | rs1444782 | A | G | 0.3777537 | -0.0257 | 0.0017 | 3.89E-52 | 0.304723889 |
|  | rs145605569 | T | C | 0.259451 | -0.018 | 0.002 | 3.85E-19 | 0.122163811 |
|  | rs145680980 | C | T | 0.0083871 | 0.064 | 0.0097 | 4.44E-11 | 0.066846455 |
|  | rs147710575 | T | C | 0.0255376 | -0.0336 | 0.0057 | 3.83E-09 | 0.055129298 |
|  | rs1479594 | G | A | 0.5253412 | -0.0113 | 0.0016 | 4.82E-12 | 0.062480124 |
|  | rs1488830 | T | C | 0.744534 | 0.0114 | 0.002 | 6.53E-09 | 0.048504625 |
|  | rs149136677 | C | G | 0.0110841 | -0.055 | 0.0085 | 1.07E-10 | 0.065065132 |
|  | rs149689913 | A | G | 0.0208269 | -0.0433 | 0.0065 | 1.94E-11 | 0.075028705 |
|  | rs149717632 | G | A | 0.22137 | 0.0237 | 0.0023 | 8.30E-26 | 0.190005 |
|  | rs150348333 | C | T | 0.0919866 | -0.0234 | 0.0035 | 1.47E-11 | 0.089747539 |
|  | rs150640087 | T | G | 0.016518 | 0.0628 | 0.007 | 4.34E-19 | 0.125728333 |
|  | rs153146 | A | G | 0.2438178 | -0.0168 | 0.0019 | 5.70E-18 | 0.102115092 |
|  | rs1533299 | T | C | 0.3820574 | -0.0103 | 0.0017 | 1.19E-09 | 0.049148164 |
|  | rs1553675 | G | A | 0.3861002 | -0.012 | 0.0018 | 2.74E-11 | 0.066976736 |
|  | rs1580991 | A | G | 0.6485329 | 0.0098 | 0.0017 | 2.09E-08 | 0.042955838 |
|  | rs1609620 | C | T | 0.4360252 | -0.0095 | 0.0016 | 8.70E-09 | 0.043548392 |
|  | rs1630916 | G | A | 0.4645084 | -0.0139 | 0.0017 | 1.64E-16 | 0.094308735 |
|  | rs1655558 | G | T | 0.5897804 | 0.011 | 0.0017 | 7.04E-11 | 0.057444952 |
|  | rs1684578 | T | G | 0.3616585 | 0.0126 | 0.0017 | 2.18E-13 | 0.07192155 |
|  | rs1689510 | C | G | 0.3082349 | 0.0154 | 0.0018 | 3.05E-18 | 0.09923393 |
|  | rs16917546 | C | T | 0.3917509 | -0.0309 | 0.0017 | 4.05E-75 | 0.446622355 |
|  | rs16975684 | A | G | 0.091499 | 0.0193 | 0.0028 | 4.72E-12 | 0.060759968 |
|  | rs17005891 | A | G | 0.2163859 | -0.0307 | 0.002 | 5.28E-51 | 0.313675893 |
|  | rs17123396 | A | G | 0.0891356 | 0.0204 | 0.003 | 8.95E-12 | 0.066302348 |
|  | rs1728780 | C | G | 0.7735862 | 0.0167 | 0.0019 | 1.16E-17 | 0.095856449 |
|  | rs17366980 | T | C | 0.1018602 | -0.0162 | 0.0026 | 6.01E-10 | 0.047112247 |
|  | rs17388568 | A | G | 0.2377342 | 0.018 | 0.0019 | 3.63E-21 | 0.11522016 |
|  | rs174583 | T | C | 0.3629427 | -0.0145 | 0.0017 | 1.40E-17 | 0.095395771 |
|  | rs17622378 | G | A | 0.3385624 | -0.0494 | 0.0018 | 2.58E-170 | 1.073472139 |
|  | rs17669032 | G | A | 0.106372 | -0.0279 | 0.0029 | 2.91E-22 | 0.145208191 |
|  | rs1775575 | A | G | 0.2428091 | -0.0111 | 0.002 | 1.52E-08 | 0.044449847 |
|  | rs17758695 | T | C | 0.0295564 | -0.0744 | 0.0052 | 1.11E-46 | 0.311630512 |
|  | rs17764723 | C | T | 0.1271378 | -0.0231 | 0.0024 | 5.12E-22 | 0.116206421 |
|  | rs17767281 | C | T | 0.4007944 | -0.0091 | 0.0017 | 4.89E-08 | 0.039024012 |
|  | rs17781972 | A | G | 0.1449636 | -0.0134 | 0.0024 | 2.47E-08 | 0.043672365 |
|  | rs17834140 | T | C | 0.0411559 | -0.0284 | 0.004 | 1.50E-12 | 0.062456693 |
|  | rs1790963 | A | C | 0.5342681 | -0.0111 | 0.0016 | 1.17E-11 | 0.060159214 |
|  | rs182492116 | C | G | 0.0241727 | -0.0465 | 0.0061 | 1.84E-14 | 0.100088153 |
|  | rs1828813 | G | A | 0.7010358 | -0.0165 | 0.0019 | 4.22E-19 | 0.111972451 |
|  | rs186380042 | C | T | 0.202266 | -0.0135 | 0.0022 | 1.03E-09 | 0.057704331 |
|  | rs1883801 | G | A | 0.1781276 | -0.0121 | 0.0021 | 1.93E-08 | 0.042059044 |
|  | rs1898671 | T | C | 0.2877268 | 0.0275 | 0.0018 | 6.29E-52 | 0.304201437 |
|  | rs191699156 | A | G | 0.0605179 | -0.0251 | 0.0037 | 1.72E-11 | 0.070288659 |
|  | rs192397873 | C | T | 0.0532631 | 0.0245 | 0.0041 | 1.95E-09 | 0.059394816 |
|  | rs192581385 | T | C | 0.0299209 | 0.0304 | 0.0052 | 4.14E-09 | 0.052636452 |
|  | rs192903278 | G | A | 0.0261091 | 0.0311 | 0.0056 | 2.32E-08 | 0.048259041 |
|  | rs197452 | T | C | 0.1407892 | -0.0176 | 0.0023 | 6.52E-14 | 0.073529459 |
|  | rs2004925 | A | G | 0.4120459 | 0.0113 | 0.0017 | 1.52E-11 | 0.060702577 |
|  | rs200630488 | T | C | 0.328445 | 0.0113 | 0.002 | 1.11E-08 | 0.055266242 |
|  | rs201193 | T | C | 0.8110563 | -0.0167 | 0.0022 | 4.06E-14 | 0.083866373 |
|  | rs2024050 | G | A | 0.895677 | -0.0361 | 0.0029 | 4.00E-36 | 0.238993459 |
|  | rs2066688 | T | C | 0.0580835 | -0.0244 | 0.0035 | 4.77E-12 | 0.063915636 |
|  | rs2067819 | A | G | 0.1760638 | 0.0176 | 0.0021 | 7.38E-17 | 0.088178445 |
|  | rs2075995 | A | C | 0.536231 | -0.0131 | 0.0016 | 1.39E-15 | 0.083746688 |
|  | rs2092856 | C | A | 0.240383 | 0.0198 | 0.0021 | 1.10E-21 | 0.140483504 |
|  | rs2111485 | G | A | 0.5193952 | -0.0122 | 0.0017 | 9.93E-13 | 0.072907517 |
|  | rs2145394 | A | G | 0.1945367 | -0.0124 | 0.0021 | 1.73E-09 | 0.047276577 |
|  | rs2147904 | C | T | 0.5575537 | 0.016 | 0.0016 | 3.52E-22 | 0.123929996 |
|  | rs2155219 | T | G | 0.4852174 | 0.0286 | 0.0016 | 2.87E-68 | 0.401055179 |
|  | rs2221641 | T | C | 0.5754625 | -0.0104 | 0.0017 | 5.98E-10 | 0.051850989 |
|  | rs2228467 | C | T | 0.0608064 | 0.0428 | 0.0037 | 1.71E-31 | 0.205313341 |
|  | rs2242886 | T | C | 0.0582372 | -0.0637 | 0.0034 | 3.16E-79 | 0.436866041 |
|  | rs2275984 | C | T | 0.2838283 | -0.0174 | 0.0018 | 5.20E-22 | 0.120770035 |
|  | rs2281525 | C | G | 0.698516 | 0.0136 | 0.0018 | 2.80E-14 | 0.076434006 |
|  | rs228978 | C | T | 0.2290691 | 0.0145 | 0.0019 | 5.05E-14 | 0.07285922 |
|  | rs2296329 | G | A | 0.1847501 | 0.0154 | 0.0021 | 2.61E-13 | 0.07009422 |
|  | rs2314338 | C | T | 0.2834114 | 0.0122 | 0.0018 | 2.96E-11 | 0.059315402 |
|  | rs2324919 | T | C | 0.2760954 | 0.0115 | 0.0018 | 2.34E-10 | 0.051867277 |
|  | rs2412771 | C | T | 0.4212995 | -0.0107 | 0.0017 | 1.52E-10 | 0.054773557 |
|  | rs2426332 | G | A | 0.568991 | -0.0101 | 0.0017 | 1.40E-09 | 0.049089719 |
|  | rs2429454 | G | T | 0.696243 | 0.0131 | 0.0019 | 9.11E-12 | 0.071218954 |
|  | rs244674 | C | T | 0.839832 | -0.0145 | 0.0024 | 1.01E-09 | 0.05549618 |
|  | rs245829 | A | T | 0.3525269 | -0.0107 | 0.0018 | 8.65E-10 | 0.051278888 |
|  | rs2471601 | A | C | 0.1509244 | 0.017 | 0.0026 | 7.84E-11 | 0.072672508 |
|  | rs2488075 | C | T | 0.416346 | -0.0187 | 0.0017 | 6.44E-28 | 0.166763584 |
|  | rs2501426 | A | G | 0.5788811 | 0.0123 | 0.0017 | 9.62E-14 | 0.072372022 |
|  | rs2509903 | C | T | 0.8830666 | 0.0162 | 0.0025 | 6.20E-11 | 0.053176516 |
|  | rs2633594 | G | A | 0.6451377 | -0.0107 | 0.0019 | 2.86E-08 | 0.051432417 |
|  | rs2646438 | A | G | 0.607406 | -0.0134 | 0.0017 | 1.61E-15 | 0.084024101 |
|  | rs2664593 | G | C | 0.2032867 | 0.0126 | 0.002 | 7.31E-10 | 0.050455528 |
|  | rs2711970 | C | T | 0.6813563 | -0.0124 | 0.0018 | 1.36E-12 | 0.06550679 |
|  | rs2810489 | A | G | 0.3163802 | -0.0153 | 0.0018 | 8.22E-17 | 0.09935395 |
|  | rs2834083 | A | G | 0.5969823 | -0.0123 | 0.0017 | 2.18E-13 | 0.071426894 |
|  | rs2847224 | A | C | 0.3666502 | 0.0178 | 0.0017 | 9.43E-25 | 0.144388907 |
|  | rs28567906 | G | A | 0.0896916 | 0.0261 | 0.0029 | 8.61E-19 | 0.109145045 |
|  | rs28595479 | C | T | 0.0309199 | 0.0285 | 0.0051 | 2.06E-08 | 0.047757651 |
|  | rs28703037 | C | G | 0.069761 | 0.0952 | 0.0088 | 4.24E-27 | 1.155384748 |
|  | rs2887944 | T | G | 0.5187991 | 0.0127 | 0.0017 | 1.18E-13 | 0.079013701 |
|  | rs2961914 | A | C | 0.2098182 | -0.0123 | 0.002 | 1.10E-09 | 0.049219423 |
|  | rs301161 | A | G | 0.7817279 | 0.0167 | 0.0022 | 9.99E-15 | 0.093381849 |
|  | rs301806 | T | C | 0.6377866 | 0.0164 | 0.0017 | 7.03E-22 | 0.121931503 |
|  | rs3024971 | G | T | 0.0913541 | -0.0276 | 0.0028 | 2.75E-23 | 0.124088105 |
|  | rs310747 | G | T | 0.6244659 | -0.0192 | 0.0017 | 1.65E-29 | 0.169656295 |
|  | rs3124998 | T | C | 0.0245315 | -0.031 | 0.0057 | 4.79E-08 | 0.045124776 |
|  | rs318095 | C | T | 0.5721187 | -0.0097 | 0.0017 | 5.59E-09 | 0.045196755 |
|  | rs322672 | C | T | 0.3108189 | 0.01 | 0.0018 | 1.41E-08 | 0.042033322 |
|  | rs34225843 | T | C | 0.0164316 | -0.0468 | 0.0076 | 7.12E-10 | 0.069461029 |
|  | rs34249860 | T | C | 0.0450936 | 0.0217 | 0.0039 | 1.77E-08 | 0.039787515 |
|  | rs34290285 | A | G | 0.2345726 | -0.0316 | 0.0019 | 1.71E-60 | 0.351921337 |
|  | rs34317837 | A | G | 0.1137698 | -0.0155 | 0.0026 | 1.54E-09 | 0.047532671 |
|  | rs34635893 | T | C | 0.1458733 | -0.0132 | 0.0023 | 8.44E-09 | 0.042598972 |
|  | rs34645101 | C | T | 0.0111646 | 0.0651 | 0.0084 | 6.84E-15 | 0.09181299 |
|  | rs346835 | T | C | 0.3158789 | -0.0188 | 0.0018 | 9.06E-27 | 0.149889073 |
|  | rs35150201 | G | T | 0.4527519 | -0.0124 | 0.0017 | 7.53E-14 | 0.0747576 |
|  | rs35576122 | G | A | 0.115631 | -0.0362 | 0.0028 | 1.28E-39 | 0.263012073 |
|  | rs35979828 | T | C | 0.0690613 | -0.0223 | 0.0035 | 1.16E-10 | 0.062737567 |
|  | rs35992154 | A | T | 0.190993 | 0.0144 | 0.0022 | 1.20E-10 | 0.062871943 |
|  | rs36034432 | A | G | 0.0758005 | -0.0199 | 0.0034 | 3.46E-09 | 0.054438029 |
|  | rs36084354 | A | G | 0.0735942 | -0.0274 | 0.003 | 1.32E-19 | 0.100444138 |
|  | rs36085856 | C | G | 0.0758432 | 0.0239 | 0.0032 | 5.43E-14 | 0.0785647 |
|  | rs374509586 | G | C | 0.672406 | 0.0145 | 0.002 | 7.42E-13 | 0.090882045 |
|  | rs3755397 | G | A | 0.0975608 | -0.0285 | 0.003 | 1.06E-21 | 0.140339357 |
|  | rs375582 | C | T | 0.7608811 | -0.0127 | 0.0019 | 2.65E-11 | 0.057583487 |
|  | rs3767748 | A | T | 0.0567309 | 0.0315 | 0.0041 | 2.94E-14 | 0.104197455 |
|  | rs3781889 | A | G | 0.2845438 | 0.0137 | 0.0018 | 7.53E-14 | 0.074979207 |
|  | rs3785356 | T | C | 0.33784 | 0.0218 | 0.0018 | 6.70E-35 | 0.208647756 |
|  | rs3790316 | G | A | 0.3951096 | 0.0133 | 0.0017 | 1.43E-15 | 0.082959978 |
|  | rs3792783 | G | A | 0.1717711 | -0.0139 | 0.0022 | 2.54E-10 | 0.053937184 |
|  | rs3802597 | G | A | 0.5640367 | -0.0142 | 0.0017 | 1.25E-17 | 0.097299676 |
|  | rs3823536 | A | G | 0.4181589 | -0.0112 | 0.0017 | 2.30E-11 | 0.059888391 |
|  | rs3824867 | G | A | 0.6806232 | 0.0139 | 0.0018 | 4.54E-15 | 0.082415815 |
|  | rs3848321 | T | A | 0.309717 | -0.015 | 0.0019 | 2.96E-15 | 0.094395407 |
|  | rs3862006 | A | G | 0.1959929 | -0.0123 | 0.0022 | 1.81E-08 | 0.046780569 |
|  | rs386869 | G | A | 0.1747493 | -0.0392 | 0.0021 | 1.28E-76 | 0.435011112 |
|  | rs389883 | T | G | 0.6784652 | 0.035 | 0.0017 | 1.93E-89 | 0.524636135 |
|  | rs412884 | C | T | 0.6197365 | 0.036 | 0.0017 | 7.49E-97 | 0.599648073 |
|  | rs41317559 | A | G | 0.0706905 | 0.0226 | 0.0032 | 1.26E-12 | 0.06584181 |
|  | rs41523455 | T | C | 0.3615551 | 0.0112 | 0.0017 | 3.99E-11 | 0.05681898 |
|  | rs42031 | T | A | 0.1764869 | 0.0191 | 0.0021 | 2.00E-19 | 0.104047132 |
|  | rs4236746 | G | A | 0.974548 | 0.0351 | 0.0056 | 3.13E-10 | 0.059965335 |
|  | rs4253766 | T | C | 0.111187 | -0.0207 | 0.0028 | 1.27E-13 | 0.083095255 |
|  | rs4367609 | T | C | 0.0656209 | 0.0316 | 0.0033 | 5.01E-22 | 0.120150912 |
|  | rs442174 | A | T | 0.0180467 | 0.0594 | 0.0073 | 5.21E-16 | 0.122701893 |
|  | rs445 | T | C | 0.1399328 | -0.0382 | 0.0025 | 6.73E-51 | 0.344719265 |
|  | rs4476815 | C | G | 0.0231527 | 0.046 | 0.0061 | 5.96E-14 | 0.093911821 |
|  | rs45458192 | T | C | 0.3761258 | -0.0111 | 0.0017 | 6.65E-11 | 0.056732974 |
|  | rs456057 | G | A | 0.886709 | 0.0166 | 0.0028 | 2.45E-09 | 0.054318915 |
|  | rs4594881 | T | G | 0.3106229 | -0.0236 | 0.0018 | 4.59E-41 | 0.234073516 |
|  | rs4645878 | G | A | 0.8997333 | 0.0194 | 0.0027 | 2.91E-13 | 0.066625063 |
|  | rs4647858 | C | T | 0.0987017 | 0.0155 | 0.0027 | 1.33E-08 | 0.041938169 |
|  | rs4675360 | T | A | 0.1317531 | -0.0254 | 0.0032 | 2.86E-15 | 0.144833831 |
|  | rs467728 | A | G | 0.273337 | -0.0115 | 0.0018 | 3.28E-10 | 0.051544736 |
|  | rs4680588 | G | T | 0.4745854 | -0.0099 | 0.0017 | 2.84E-09 | 0.047955946 |
|  | rs474247 | T | C | 0.2540737 | 0.0143 | 0.002 | 6.14E-13 | 0.07604938 |
|  | rs4758576 | A | G | 0.804087 | -0.0141 | 0.002 | 4.18E-12 | 0.061456261 |
|  | rs478839 | G | A | 0.3989689 | 0.0176 | 0.0017 | 1.03E-25 | 0.145767327 |
|  | rs4802399 | A | G | 0.0703164 | 0.0235 | 0.0035 | 1.72E-11 | 0.070842348 |
|  | rs4822027 | A | G | 0.1764069 | 0.0189 | 0.0021 | 3.24E-19 | 0.101843049 |
|  | rs4857909 | G | A | 0.8354387 | 0.0815 | 0.0024 | 1.00E-200 | 1.795087984 |
|  | rs4871849 | A | G | 0.710139 | -0.0226 | 0.0019 | 3.68E-31 | 0.206336328 |
|  | rs4889539 | T | G | 0.0442773 | 0.0272 | 0.0044 | 7.08E-10 | 0.061434506 |
|  | rs4939489 | T | C | 0.3721812 | 0.0096 | 0.0017 | 1.24E-08 | 0.042255599 |
|  | rs4970966 | T | G | 0.2147827 | -0.0293 | 0.002 | 4.55E-49 | 0.284174126 |
|  | rs503734 | G | A | 0.4464826 | -0.0111 | 0.0017 | 2.56E-11 | 0.059750641 |
|  | rs531660643 | T | G | 0.0232735 | -0.0504 | 0.006 | 4.35E-17 | 0.113313131 |
|  | rs534977154 | C | T | 0.0604459 | -0.0219 | 0.0039 | 2.59E-08 | 0.05344842 |
|  | rs553772010 | G | A | 0.114904 | -0.025 | 0.0028 | 2.13E-19 | 0.124736943 |
|  | rs55909515 | T | G | 0.0335281 | -0.0354 | 0.0049 | 4.20E-13 | 0.079684584 |
|  | rs559100991 | T | C | 0.1054877 | 0.0257 | 0.0027 | 6.05E-21 | 0.1223046 |
|  | rs55977204 | C | T | 0.1574022 | 0.014 | 0.0024 | 4.85E-09 | 0.051008691 |
|  | rs56076326 | T | C | 0.2273264 | 0.0128 | 0.0019 | 5.17E-11 | 0.05647096 |
|  | rs56130935 | G | A | 0.3945594 | 0.0109 | 0.0017 | 1.02E-10 | 0.055692401 |
|  | rs56189237 | A | G | 0.1133294 | 0.0217 | 0.0025 | 1.09E-17 | 0.092853826 |
|  | rs56274618 | A | G | 0.0097813 | 0.0612 | 0.0091 | 1.59E-11 | 0.071186008 |
|  | rs56320921 | T | C | 0.0702103 | -0.0189 | 0.0034 | 4.33E-08 | 0.045757655 |
|  | rs56375023 | A | G | 0.1963916 | 0.02 | 0.002 | 6.62E-23 | 0.123884378 |
|  | rs56408717 | A | G | 0.1381157 | 0.0178 | 0.0024 | 1.08E-13 | 0.07401149 |
|  | rs567910619 | T | C | 0.0108605 | -0.0544 | 0.0089 | 1.20E-09 | 0.062383124 |
|  | rs56949365 | C | T | 0.4258847 | 0.0152 | 0.0017 | 2.66E-20 | 0.110856645 |
|  | rs570765844 | C | T | 0.0805242 | 0.0244 | 0.0033 | 8.25E-14 | 0.086500577 |
|  | rs57453899 | A | G | 0.2767974 | -0.0138 | 0.002 | 1.57E-12 | 0.074807923 |
|  | rs5746451 | C | T | 0.57877 | 0.0098 | 0.0017 | 6.79E-09 | 0.045944353 |
|  | rs575638 | T | C | 0.4196592 | 0.0204 | 0.0017 | 6.25E-34 | 0.198912727 |
|  | rs58745116 | A | G | 0.3243929 | -0.0099 | 0.0018 | 1.87E-08 | 0.042149162 |
|  | rs59322 | A | C | 0.46392 | -0.0093 | 0.0016 | 1.70E-08 | 0.042207693 |
|  | rs59367227 | C | T | 0.0463575 | -0.0252 | 0.0045 | 1.49E-08 | 0.055089116 |
|  | rs59741680 | T | C | 0.1779706 | -0.0158 | 0.0023 | 8.74E-12 | 0.071666432 |
|  | rs59849941 | G | A | 0.141074 | -0.0193 | 0.003 | 1.56E-10 | 0.08857087 |
|  | rs60175411 | A | G | 0.1933204 | -0.0214 | 0.0021 | 1.03E-23 | 0.140153075 |
|  | rs6031304 | C | T | 0.7304659 | -0.015 | 0.0018 | 3.90E-16 | 0.086929863 |
|  | rs6037542 | G | T | 0.8427507 | 0.0149 | 0.0024 | 7.37E-10 | 0.057732486 |
|  | rs604255 | T | G | 0.1943255 | 0.0156 | 0.002 | 2.60E-14 | 0.074766319 |
|  | rs60645096 | T | C | 0.0287832 | 0.0405 | 0.006 | 1.11E-11 | 0.089978648 |
|  | rs61033457 | A | G | 0.073397 | -0.029 | 0.0045 | 7.70E-11 | 0.112241175 |
|  | rs61048992 | T | C | 0.0546033 | 0.0191 | 0.0035 | 4.02E-08 | 0.036953054 |
|  | rs61096966 | T | C | 0.6986923 | 0.0258 | 0.0018 | 1.58E-47 | 0.27503737 |
|  | rs6126029 | A | C | 0.0820964 | -0.0212 | 0.003 | 1.00E-12 | 0.06645944 |
|  | rs61612642 | T | C | 0.164257 | 0.0241 | 0.0024 | 7.23E-24 | 0.156471166 |
|  | rs61865678 | A | G | 0.1176509 | 0.0154 | 0.0028 | 3.02E-08 | 0.048309532 |
|  | rs61953394 | C | T | 0.1279268 | 0.018 | 0.0026 | 5.62E-12 | 0.070929225 |
|  | rs61975764 | A | G | 0.465326 | 0.0147 | 0.0018 | 1.15E-16 | 0.105502341 |
|  | rs62037086 | A | G | 0.1449028 | -0.0141 | 0.0023 | 1.34E-09 | 0.048337731 |
|  | rs62061733 | G | A | 0.228862 | -0.0214 | 0.0021 | 1.72E-24 | 0.158612674 |
|  | rs62066853 | C | T | 0.2654201 | 0.0124 | 0.0019 | 9.39E-11 | 0.058826979 |
|  | rs62096277 | C | T | 0.2106925 | 0.0128 | 0.002 | 3.73E-10 | 0.053465448 |
|  | rs62375214 | C | G | 0.0906168 | 0.0172 | 0.0031 | 2.04E-08 | 0.047837454 |
|  | rs62541534 | G | C | 0.2574095 | 0.0211 | 0.0019 | 1.94E-29 | 0.167011779 |
|  | rs6440573 | G | C | 0.9523401 | -0.0209 | 0.0038 | 4.45E-08 | 0.038903557 |
|  | rs6441620 | A | C | 0.5757428 | 0.014 | 0.0017 | 7.45E-17 | 0.093948475 |
|  | rs6496712 | T | G | 0.7411316 | 0.0135 | 0.0019 | 1.01E-12 | 0.068613035 |
|  | rs6535847 | C | T | 0.6529146 | -0.0121 | 0.0018 | 1.04E-11 | 0.065106838 |
|  | rs6556313 | G | A | 0.3359707 | 0.015 | 0.0017 | 5.77E-18 | 0.098502927 |
|  | rs6559744 | A | G | 0.2555817 | 0.0111 | 0.0019 | 3.04E-09 | 0.045998901 |
|  | rs6580223 | T | G | 0.6195073 | 0.0248 | 0.0017 | 3.07E-49 | 0.284548565 |
|  | rs6580586 | C | A | 0.1119479 | 0.0183 | 0.0026 | 3.27E-12 | 0.065331068 |
|  | rs6589563 | A | G | 0.1089476 | -0.0187 | 0.0028 | 2.04E-11 | 0.066614391 |
|  | rs6598043 | C | G | 0.6653619 | -0.0205 | 0.0018 | 1.10E-30 | 0.183635513 |
|  | rs6672038 | T | C | 0.1645806 | 0.0269 | 0.0024 | 2.95E-30 | 0.19525789 |
|  | rs6682237 | C | A | 0.1588659 | 0.0194 | 0.0024 | 1.51E-16 | 0.098690963 |
|  | rs67121524 | C | T | 0.1935759 | -0.0122 | 0.0022 | 1.69E-08 | 0.045592035 |
|  | rs6739447 | A | G | 0.4785236 | 0.0102 | 0.0016 | 6.37E-10 | 0.050944259 |
|  | rs6753667 | A | G | 0.6855608 | 0.0288 | 0.0018 | 9.92E-60 | 0.350959736 |
|  | rs67760360 | A | G | 0.2463048 | -0.0192 | 0.0019 | 2.48E-24 | 0.134296547 |
|  | rs6787336 | A | G | 0.2556695 | 0.0205 | 0.0019 | 3.13E-27 | 0.156948185 |
|  | rs67901225 | C | T | 0.1486534 | -0.0143 | 0.0024 | 4.39E-09 | 0.050782045 |
|  | rs6793085 | T | C | 0.4699119 | -0.0106 | 0.0016 | 1.33E-10 | 0.05492055 |
|  | rs6798870 | A | G | 0.553064 | 0.0103 | 0.0017 | 7.48E-10 | 0.051457927 |
|  | rs6801612 | G | A | 0.2098037 | 0.0112 | 0.0021 | 4.48E-08 | 0.040807186 |
|  | rs6852559 | A | G | 0.2544433 | 0.0145 | 0.0019 | 5.81E-14 | 0.078266644 |
|  | rs686024 | T | C | 0.0566313 | 0.0224 | 0.0038 | 3.88E-09 | 0.052600716 |
|  | rs6884604 | C | T | 0.0742987 | 0.0196 | 0.003 | 1.03E-10 | 0.051846752 |
|  | rs6884762 | T | C | 0.0249464 | 0.0551 | 0.0058 | 1.03E-21 | 0.144923248 |
|  | rs6904506 | C | T | 0.1491768 | -0.0301 | 0.0026 | 6.38E-31 | 0.225687614 |
|  | rs6932056 | C | T | 0.0399669 | -0.0292 | 0.0044 | 2.28E-11 | 0.064197048 |
|  | rs6932503 | A | G | 0.4946056 | 0.0104 | 0.0016 | 2.66E-10 | 0.05305349 |
|  | rs699664 | T | C | 0.3231429 | -0.0112 | 0.0017 | 1.17E-10 | 0.053837614 |
|  | rs7013461 | T | A | 0.2536289 | 0.0126 | 0.0019 | 6.29E-11 | 0.058973255 |
|  | rs7018860 | A | G | 0.5399192 | -0.0104 | 0.0017 | 8.24E-10 | 0.052721437 |
|  | rs7020893 | A | G | 0.2280531 | -0.0208 | 0.002 | 1.31E-24 | 0.149468806 |
|  | rs706778 | T | C | 0.4361 | 0.0117 | 0.0017 | 1.60E-12 | 0.066057705 |
|  | rs7080536 | A | G | 0.0439352 | -0.0287 | 0.0044 | 4.58E-11 | 0.067893488 |
|  | rs71355361 | A | G | 0.1243142 | 0.0153 | 0.0025 | 5.17E-10 | 0.050004427 |
|  | rs71368508 | A | C | 0.0210665 | -0.1176 | 0.0062 | 6.48E-81 | 0.559941118 |
|  | rs71505834 | G | A | 0.299743 | 0.0112 | 0.0019 | 5.86E-09 | 0.051665396 |
|  | rs7206884 | A | G | 0.5658242 | -0.0104 | 0.0016 | 2.87E-10 | 0.052140024 |
|  | rs7214671 | C | G | 0.0329273 | 0.0304 | 0.0056 | 4.43E-08 | 0.057746086 |
|  | rs725613 | G | T | 0.3185522 | -0.0322 | 0.0017 | 8.05E-76 | 0.441829633 |
|  | rs7258283 | T | C | 0.3153073 | 0.0141 | 0.0018 | 7.34E-16 | 0.084224714 |
|  | rs7275212 | T | A | 0.0247051 | 0.042 | 0.0061 | 3.84E-12 | 0.083405213 |
|  | rs72755295 | G | A | 0.0334515 | -0.0286 | 0.0049 | 5.59E-09 | 0.051895372 |
|  | rs72836561 | T | C | 0.0318217 | -0.0382 | 0.005 | 2.34E-14 | 0.088222394 |
|  | rs72837826 | T | G | 0.102361 | 0.0364 | 0.0029 | 4.49E-36 | 0.238934629 |
|  | rs72899879 | G | A | 0.129743 | 0.0161 | 0.0027 | 3.95E-09 | 0.05743055 |
|  | rs72901762 | G | A | 0.2538794 | -0.0123 | 0.0019 | 4.54E-11 | 0.056234901 |
|  | rs72922276 | A | G | 0.111754 | -0.026 | 0.0028 | 2.03E-20 | 0.131684804 |
|  | rs7302975 | C | T | 0.8026551 | -0.0155 | 0.002 | 2.53E-14 | 0.074676781 |
|  | rs73072483 | A | G | 0.1387431 | -0.0243 | 0.0024 | 5.14E-24 | 0.138468894 |
|  | rs7314285 | G | T | 0.0813374 | -0.0243 | 0.0031 | 3.25E-15 | 0.086582774 |
|  | rs73205398 | T | C | 0.0746572 | 0.025 | 0.003 | 2.03E-16 | 0.084727885 |
|  | rs73207326 | G | A | 0.4114636 | 0.0095 | 0.0017 | 1.36E-08 | 0.042884988 |
|  | rs73240305 | G | A | 0.1537907 | -0.014 | 0.0023 | 5.21E-10 | 0.050051891 |
|  | rs733210 | G | A | 0.1856483 | 0.0134 | 0.0022 | 5.38E-10 | 0.053268498 |
|  | rs7337995 | G | A | 0.3752081 | 0.0247 | 0.0018 | 7.99E-43 | 0.280711432 |
|  | rs73455661 | G | A | 0.2670745 | 0.0105 | 0.0018 | 1.03E-08 | 0.042347129 |
|  | rs7423615 | T | C | 0.18738 | 0.0156 | 0.0022 | 3.71E-12 | 0.072715411 |
|  | rs74253035 | T | G | 0.0701647 | 0.0284 | 0.0041 | 7.40E-12 | 0.103262186 |
|  | rs74480102 | A | G | 0.0431706 | -0.0533 | 0.0043 | 5.76E-35 | 0.230310264 |
|  | rs74612091 | A | T | 0.0635639 | 0.0393 | 0.0036 | 2.09E-27 | 0.180421373 |
|  | rs74625348 | C | G | 0.2544222 | 0.0159 | 0.0019 | 2.95E-16 | 0.094106207 |
|  | rs74697736 | A | G | 0.2787957 | -0.0126 | 0.0018 | 4.30E-12 | 0.062639393 |
|  | rs74752715 | G | A | 0.0923566 | 0.0163 | 0.0028 | 6.82E-09 | 0.04370307 |
|  | rs7503461 | T | C | 0.3387713 | -0.0098 | 0.0017 | 1.61E-08 | 0.042214677 |
|  | rs7523334 | A | G | 0.5180872 | 0.0099 | 0.0017 | 1.93E-09 | 0.048017253 |
|  | rs75480723 | G | A | 0.0094807 | -0.0515 | 0.0091 | 1.53E-08 | 0.04887364 |
|  | rs7554373 | G | T | 0.2463254 | 0.0144 | 0.0019 | 2.69E-14 | 0.075541535 |
|  | rs75639632 | G | A | 0.033666 | 0.083 | 0.005 | 1.50E-62 | 0.4399507 |
|  | rs7602700 | G | T | 0.364847 | -0.0111 | 0.0017 | 6.08E-11 | 0.056026595 |
|  | rs7617986 | C | G | 0.0321996 | -0.0351 | 0.0047 | 5.86E-14 | 0.075338674 |
|  | rs7646283 | T | C | 0.2976651 | 0.0165 | 0.0018 | 8.85E-20 | 0.11169245 |
|  | rs76474320 | C | T | 0.0972226 | 0.0214 | 0.0029 | 1.22E-13 | 0.078875989 |
|  | rs76633497 | C | T | 0.0856593 | -0.0254 | 0.003 | 2.54E-17 | 0.099158118 |
|  | rs766814 | A | G | 0.3006044 | 0.0166 | 0.0018 | 3.88E-20 | 0.113689167 |
|  | rs76720453 | C | T | 0.062389 | 0.0327 | 0.0037 | 2.52E-18 | 0.122748133 |
|  | rs76883015 | A | T | 0.0602346 | -0.0254 | 0.0039 | 4.82E-11 | 0.071663659 |
|  | rs7688870 | C | A | 0.4577776 | 0.0099 | 0.0016 | 1.44E-09 | 0.0477373 |
|  | rs76919997 | C | G | 0.023275 | -0.0455 | 0.0058 | 7.27E-15 | 0.092354914 |
|  | rs77050486 | A | C | 0.0313782 | -0.038 | 0.0051 | 7.45E-14 | 0.08612355 |
|  | rs7746061 | A | G | 0.071485 | 0.1044 | 0.0087 | 4.74E-33 | 1.421569225 |
|  | rs77665773 | T | C | 0.0596977 | 0.023 | 0.0037 | 7.92E-10 | 0.058269448 |
|  | rs7778729 | C | T | 0.253674 | 0.0132 | 0.0019 | 1.28E-12 | 0.06473146 |
|  | rs778756 | G | A | 0.5419053 | -0.0108 | 0.0017 | 7.93E-11 | 0.056817965 |
|  | rs7845819 | T | C | 0.1740283 | 0.0134 | 0.0022 | 1.08E-09 | 0.05064672 |
|  | rs789862 | G | A | 0.3359687 | -0.0102 | 0.0018 | 5.83E-09 | 0.045545158 |
|  | rs79036644 | C | G | 0.0147925 | -0.0429 | 0.0074 | 6.23E-09 | 0.052630986 |
|  | rs7903956 | A | T | 0.296521 | -0.0115 | 0.0018 | 2.45E-10 | 0.054132809 |
|  | rs7939912 | A | G | 0.6040487 | 0.0105 | 0.0017 | 4.79E-10 | 0.051742756 |
|  | rs79453306 | G | A | 0.2405893 | -0.0114 | 0.0019 | 1.64E-09 | 0.046592675 |
|  | rs796904 | G | T | 0.2755382 | -0.0157 | 0.0018 | 7.04E-18 | 0.096554754 |
|  | rs79701703 | T | C | 0.0310785 | 0.0319 | 0.0051 | 2.97E-10 | 0.060129967 |
|  | rs79716587 | A | G | 0.1059973 | -0.0152 | 0.0026 | 6.82E-09 | 0.042960991 |
|  | rs79774308 | G | A | 0.0207361 | 0.0386 | 0.0065 | 2.28E-09 | 0.059369398 |
|  | rs7980311 | T | C | 0.4251047 | -0.0102 | 0.0017 | 7.41E-10 | 0.049893207 |
|  | rs79926321 | T | C | 0.0135899 | -0.0539 | 0.0076 | 1.66E-12 | 0.076422188 |
|  | rs80001447 | T | A | 0.0575338 | -0.0236 | 0.0039 | 9.39E-10 | 0.059261607 |
|  | rs80014247 | G | T | 0.0299958 | 0.0305 | 0.0053 | 7.66E-09 | 0.053111882 |
|  | rs80054178 | C | T | 0.0224364 | -0.0518 | 0.0059 | 2.90E-18 | 0.115489679 |
|  | rs80163833 | T | C | 0.2200294 | 0.024 | 0.0021 | 9.94E-30 | 0.193999951 |
|  | rs80228232 | T | C | 0.0329976 | 0.0386 | 0.0054 | 6.85E-13 | 0.093295315 |
|  | rs80230740 | C | T | 0.0175039 | -0.0537 | 0.0071 | 2.92E-14 | 0.097317658 |
|  | rs80323277 | T | C | 0.009483 | -0.0573 | 0.0095 | 1.38E-09 | 0.060517156 |
|  | rs8051988 | T | C | 0.2252816 | -0.0142 | 0.0019 | 2.25E-13 | 0.069057555 |
|  | rs8068894 | G | A | 0.0345689 | -0.0442 | 0.0048 | 4.26E-20 | 0.12795061 |
|  | rs8142988 | C | T | 0.4147972 | -0.0137 | 0.0017 | 2.74E-15 | 0.089404062 |
|  | rs844648 | A | G | 0.4148427 | 0.0124 | 0.0017 | 8.77E-14 | 0.073243015 |
|  | rs900382 | T | C | 0.1087538 | -0.03 | 0.0035 | 2.16E-17 | 0.171196454 |
|  | rs912131 | G | A | 0.6476012 | 0.0171 | 0.0018 | 4.45E-22 | 0.130956351 |
|  | rs913423 | A | G | 0.5696097 | 0.0109 | 0.0017 | 5.88E-11 | 0.057154771 |
|  | rs9271790 | A | G | 0.192505 | -0.0307 | 0.0021 | 1.91E-50 | 0.287554568 |
|  | rs9308628 | T | C | 0.2406933 | -0.0182 | 0.0019 | 3.77E-21 | 0.118798504 |
|  | rs934137 | C | T | 0.5513641 | 0.0188 | 0.0016 | 4.87E-30 | 0.171576771 |
|  | rs9357149 | T | C | 0.0185962 | 0.066 | 0.0065 | 4.70E-24 | 0.156013871 |
|  | rs9357489 | C | G | 0.3550208 | 0.0102 | 0.0018 | 1.48E-08 | 0.046747123 |
|  | rs9380234 | A | G | 0.174093 | -0.0654 | 0.0058 | 3.99E-29 | 1.208194334 |
|  | rs9389268 | G | A | 0.2782191 | -0.0334 | 0.0018 | 3.08E-73 | 0.439758487 |
|  | rs9400975 | T | C | 0.6664953 | -0.0109 | 0.0017 | 4.12E-10 | 0.051821424 |
|  | rs9405665 | T | C | 0.4452559 | 0.0141 | 0.0017 | 4.16E-17 | 0.096364617 |
|  | rs9504361 | G | A | 0.4005272 | -0.0177 | 0.0017 | 6.56E-25 | 0.147620858 |
|  | rs9548932 | T | G | 0.6703294 | 0.0173 | 0.0017 | 1.54E-23 | 0.129793359 |
|  | rs9557201 | G | A | 0.2516505 | 0.0125 | 0.0019 | 2.66E-11 | 0.057740723 |
|  | rs9814893 | T | C | 0.4408556 | -0.0137 | 0.0017 | 3.93E-16 | 0.090789578 |
|  | rs9815874 | T | C | 0.2407315 | 0.0192 | 0.0019 | 8.53E-24 | 0.132228075 |
|  | rs9819371 | T | C | 0.0644251 | -0.0197 | 0.0036 | 3.54E-08 | 0.045900855 |
|  | rs9829778 | A | G | 0.4312652 | 0.01 | 0.0017 | 1.32E-09 | 0.048129336 |
|  | rs9858954 | G | A | 0.273962 | 0.0117 | 0.0019 | 2.43E-10 | 0.053429285 |
|  | rs9906532 | T | C | 0.452021 | -0.0106 | 0.0017 | 3.20E-10 | 0.05461259 |
|  | rs9916216 | A | G | 0.209772 | -0.0125 | 0.002 | 4.38E-10 | 0.050824927 |
|  | rs9920592 | T | C | 0.1558423 | -0.0171 | 0.0023 | 1.43E-13 | 0.075486437 |
|  | rs9949696 | C | T | 0.6928203 | 0.011 | 0.0018 | 2.05E-09 | 0.050530688 |
| Sum |  |  |  |  |  |  |  | **55.98980764** |

Supplementary Table 5. Association between moderate to severe asthma and AD adjusted for BMI, diabetes, hypertension, cigarettes smoked per day or total cholesterol in multivariable MR analysis.

| **Risk factor for adjustment** | **beta** | **se** | **p- value** | **OR** | **95% CI** |
| --- | --- | --- | --- | --- | --- |
| Moderate to severe asthma | 0.0416 | 0.0145 | 4.20E-03 | 1.04 | 1.01-1.07 |
| Body mass index | -0.0069 | 0.0487 | 8.88E-01 | 0.99 | 0.90-1.09 |
| Diabetes | -0.0177 | 0.0151 | 2.41E-01 | 0.98 | 0.95-1.01 |
| Hypertension | 0.2043 | 0.1283 | 1.11E-01 | 1.23 | 0.95-1.58 |
| Cigarettes smoked per day | -0.0962 | 0.0711 | 1.76E-01 | 0.91 | 0.70-1.04 |
| Total cholesterol | 0.2513 | 0.0281 | 3.52E-19 | 1.29 | 1.22-1.36 |

Supplementary Table 6. Reverse two-step MR association between AD and moderate to severe asthma.

| **Exposure** | **Outcome** | **Method** | **Number of SNPs** | **beta** | **se** | **OR** | **95%CI** | **p-value** | **p-value for Cochran Q test** | **p-value for MR-Egger intercept** | **p-value for MR-PRESSO Global test** | **p-value for MR-PRESSO Distortion Test** |
| --- | --- | --- | --- | --- | --- | --- | --- | --- | --- | --- | --- | --- |
| Alzheimer’s disease | Moderate to severe asthma | MR-Egger | 6 | 0.563 | 1.389 | 1.755 | 0.115 - 26.694 | 0.706 | 1.404830e-09 | 0.2075289 | 0.001 | 0.176 |
|  |  | Weighted median |  | -0.020 | 0.334 | 0.980 | 0.511 - 1.877 | 0.951 |  |  |  |  |
|  |  | Inverse variance weighted |  | -0.808 | 1.171 | 0.446 | 0.045 - 4.422 | 0.490 | 1.677538e-14 |  |  |  |

Supplementary Table 7. Information of genetic instrumental variants associated with inflammatory within 100 kb windows from gene FPR1, IL1RAP, IL7R or IL18RAP.

| **SNP** | **Chromosome** | **Base pair** | **Effect allele** | **Other allele** | **Effect allele frequency** | **beta** | **se** | **p-value** | **F-statistic** |
| --- | --- | --- | --- | --- | --- | --- | --- | --- | --- |
| FPR1 |  |  |  |  |  |  |  |  |  |
| rs10422101 | 19 | 52320115 | C | G | 0.270538 | -0.012777 | 0.002234 | 1.10E-08 | 3.30356086 |
| rs11667325 | 19 | 52301063 | A | G | 0.663846 | -0.018204 | 0.002098 | 4.24E-18 | 7.583489796 |
| rs58018258 | 19 | 52292898 | T | C | 0.353004 | -0.012856 | 0.002076 | 6.14E-10 | 3.870716183 |
| rs62108948 | 19 | 52311527 | C | A | 0.130163 | 0.026268 | 0.002961 | 7.62E-19 | 8.011482605 |
| rs62110082 | 19 | 52338641 | C | T | 0.046973 | 0.030885 | 0.00469 | 4.68E-11 | 4.378762559 |
| rs7251137 | 19 | 52276665 | A | C | 0.604532 | -0.014451 | 0.002039 | 1.42E-12 | 5.119592584 |
| rs7251352 | 19 | 52276704 | A | G | 0.342811 | -0.011863 | 0.002119 | 2.20E-08 | 3.251076771 |
| rs7254007 | 19 | 52180369 | G | A | 0.299338 | -0.014456 | 0.002171 | 2.89E-11 | 4.49438588 |
| rs75810459 | 19 | 52160790 | A | G | 0.087761 | -0.022082 | 0.003497 | 2.78E-10 | 4.003008466 |
| rs78773207 | 19 | 52296778 | T | G | 0.048925 | 0.030401 | 0.004623 | 5.02E-11 | 4.409854742 |
| rs885266 | 19 | 52148804 | C | A | 0.351714 | -0.014571 | 0.00227 | 1.42E-10 | 4.964124389 |
| Sum |  |  |  |  |  |  |  |  | **53.39005484** |
| IL1RAP |  |  |  |  |  |  |  |  |  |
| rs1024949 | 3 | 190347732 | C | T | 0.13439 | -0.4479 | 0.0399 | 2.95E-29 | 5.522335111 |
| rs10937442 | 3 | 190294208 | C | G | 0.4616 | -0.1879 | 0.0249 | 4.68E-14 | 2.014772854 |
| rs13071108 | 3 | 190355651 | C | T | 0.28119 | 0.5399 | 0.0267 | 8.91E-91 | 15.06617006 |
| rs13084342 | 3 | 190449374 | T | A | 0.22994 | 0.2441 | 0.0294 | 1.15E-16 | 2.43136042 |
| rs143223524 | 3 | 190415546 | C | T | 0.01556 | 0.9952 | 0.1016 | 1.17E-22 | 3.529504209 |
| rs144560286 | 3 | 190523281 | C | G | 0.0172 | 0.9456 | 0.0992 | 1.51E-21 | 3.516028043 |
| rs1466889 | 3 | 190567208 | A | G | 0.20497 | 0.1977 | 0.0339 | 5.75E-09 | 1.455350108 |
| rs148996090 | 3 | 190347247 | A | C | 0.01018 | 1.1107 | 0.1365 | 3.98E-16 | 2.875701411 |
| rs17515252 | 3 | 190452195 | G | A | 0.04363 | 0.8888 | 0.058 | 5.89E-53 | 7.96067657 |
| rs2885370 | 3 | 190276995 | T | G | 0.74273 | -0.2086 | 0.0306 | 8.91E-12 | 1.907410104 |
| rs34845858 | 3 | 190313439 | A | G | 0.02345 | 1.1625 | 0.0801 | 9.55E-48 | 7.441906219 |
| rs35335747 | 3 | 190470025 | T | C | 0.01323 | 0.9888 | 0.1189 | 9.12E-17 | 2.954854011 |
| rs36063565 | 3 | 190531903 | T | C | 0.03934 | 0.7522 | 0.0694 | 2.34E-27 | 5.039242299 |
| rs3773954 | 3 | 190314468 | T | C | 0.49935 | -0.3835 | 0.0237 | 7.08E-59 | 8.95269945 |
| rs3935774 | 3 | 190360571 | T | C | 0.01707 | 0.7411 | 0.1119 | 3.55E-11 | 2.117879633 |
| rs4686554 | 3 | 190273818 | T | C | 0.73755 | -0.1935 | 0.028 | 4.90E-12 | 1.659028574 |
| rs4687154 | 3 | 190304172 | C | G | 0.89286 | -0.581 | 0.0396 | 7.94E-49 | 7.787436029 |
| rs57370730 | 3 | 190480033 | T | A | 0.20123 | -0.2369 | 0.0308 | 1.55E-14 | 2.072354449 |
| rs60476695 | 3 | 190337290 | T | C | 0.21378 | -0.2868 | 0.0319 | 2.34E-19 | 3.207447083 |
| rs62286265 | 3 | 190378927 | G | A | 0.03834 | -0.4377 | 0.0646 | 1.20E-11 | 1.616286779 |
| rs6444439 | 3 | 190319668 | C | G | 0.97581 | -1.3591 | 0.0826 | 8.91E-61 | 10.77562668 |
| rs67249092 | 3 | 190357571 | G | A | 0.15199 | -0.379 | 0.0345 | 5.25E-28 | 4.337036389 |
| rs6763761 | 3 | 190352514 | T | G | 0.77952 | -0.9935 | 0.0257 | 1.00E-200 | 57.92010203 |
| rs6801017 | 3 | 190295730 | G | A | 0.21399 | -0.2023 | 0.0306 | 3.80E-11 | 1.57451348 |
| rs71310871 | 3 | 190286188 | G | A | 0.02355 | 0.4596 | 0.0825 | 2.51E-08 | 1.106504504 |
| rs71310875 | 3 | 190361151 | T | G | 0.065 | 0.9701 | 0.0494 | 1.02E-85 | 14.56894137 |
| rs73200020 | 3 | 190312370 | G | C | 0.06824 | -0.3292 | 0.0567 | 6.31E-09 | 1.576166447 |
| rs7628250 | 3 | 190308527 | A | G | 0.77645 | -0.8091 | 0.0263 | 1.00E-200 | 33.17199498 |
| rs78129851 | 3 | 190374933 | A | G | 0.01109 | 0.7929 | 0.1167 | 1.07E-11 | 1.577132433 |
| Sum |  |  |  |  |  |  |  |  | **215.7364617** |
| IL7R |  |  |  |  |  |  |  |  |  |
| rs3822731 | 5 | 35875240 | G | A | 0.14314 | 0.2003 | 0.0353 | 1.45E-08 | 6.550032969 |
| rs6451229 | 5 | 35866218 | G | A | 0.40041 | 0.2941 | 0.0247 | 1.29E-32 | 28.555317 |
| rs700176 | 5 | 35930703 | T | C | 0.41085 | 0.1872 | 0.025 | 6.76E-14 | 11.37279576 |
| rs852243 | 5 | 35923830 | A | T | 0.39467 | 0.1579 | 0.0249 | 2.51E-10 | 7.945308993 |
| rs931555 | 5 | 35803577 | T | C | 0.33045 | -0.1995 | 0.026 | 1.55E-14 | 11.81426909 |
| Sum |  |  |  |  |  |  |  |  | **66.23772381** |
| IL18RAP |  |  |  |  |  |  |  |  |  |
| rs10172116 | 2 | 103087573 | T | C | 0.17745 | -0.217 | 0.0326 | 2.88E-11 | 3.05241283 |
| rs114003263 | 2 | 103002194 | A | G | 0.01741 | 0.7564 | 0.1064 | 1.20E-12 | 4.372541423 |
| rs12987260 | 2 | 103055634 | T | G | 0.0362 | -0.5252 | 0.0642 | 2.69E-16 | 4.297932848 |
| rs13014644 | 2 | 102971363 | T | G | 0.10301 | -0.5629 | 0.0394 | 3.24E-46 | 13.62098024 |
| rs13385391 | 2 | 103156274 | T | C | 0.06545 | -0.4335 | 0.0499 | 3.55E-18 | 5.153058357 |
| rs145903188 | 2 | 103071259 | C | T | 0.01651 | -0.5825 | 0.1067 | 4.79E-08 | 2.440029105 |
| rs6543124 | 2 | 102987459 | A | T | 0.37861 | -0.2722 | 0.0255 | 1.62E-26 | 7.910749796 |
| rs6543139 | 2 | 103066235 | A | G | 0.93858 | -0.7816 | 0.0526 | 5.25E-50 | 16.59372645 |
| rs6543144 | 2 | 103092575 | G | A | 0.31481 | -0.4085 | 0.0262 | 9.12E-55 | 16.98889868 |
| rs74342136 | 2 | 103047986 | T | C | 0.04477 | 0.6782 | 0.0623 | 1.20E-27 | 8.968406353 |
| rs74909818 | 2 | 102938428 | G | A | 0.01949 | 0.7919 | 0.0948 | 6.61E-17 | 5.377924165 |
| rs7579737 | 2 | 102987361 | G | A | 0.30893 | -0.4228 | 0.026 | 2.69E-59 | 18.09706993 |
| rs78123896 | 2 | 103075417 | C | T | 0.14218 | -0.3866 | 0.0349 | 1.41E-28 | 8.28631938 |
| rs78756788 | 2 | 103050569 | C | T | 0.04659 | 0.7039 | 0.0577 | 3.47E-34 | 10.08366628 |
| rs79463214 | 2 | 103090575 | C | A | 0.01932 | 0.7655 | 0.0916 | 6.31E-17 | 4.973375159 |
| Sum |  |  |  |  |  |  |  |  | **130.217091** |

Supplementary Table 8. SMR association between expression of gene FPR1, IL1RAP, IL7R, or IL18RAP and AD outcomes.

| **Gene** | **Gene chromosome** | **Probe** | **Gene base pair** | **top eQTL SNP** | **SNP Chromosome** | **SNP base pair** | **Effect allele** | **Other allele** | **Effect allele frequency** | **eQTL association** | | | | **GWAS association** | | | **SMR association** | | | **HEIDI Test** | |
| --- | --- | --- | --- | --- | --- | --- | --- | --- | --- | --- | --- | --- | --- | --- | --- | --- | --- | --- | --- | --- | --- |
|  |  |  |  |  |  |  |  |  |  | **beta** | **se** | **p-value** | **F-statistic** | **beta** | **se** | **p-value** | **beta** | **se** | **p-value** | **p-value** | **Number of SNPs** |
| FPR1 | 19 | ENSG00000171051 | 52277894 | rs6509570 | 19 | 52258019 | T | C | 0.338446 | -0.4844 | 0.0081 | 0.00E+00 | 309.87 | 0.0073 | 0.0024 | 2.19E-03 | -0.0150 | 0.0049 | 2.22E-03 | 0.0013 | 20 |
| IL1RAP | 3 | ENSG00000196083 | 190303841 | rs78888631 | 3 | 190284555 | A | G | 0.0674847 | 0.3867 | 0.0159 | 4.42E-130 | 202.57 | -0.0121 | 0.0042 | 3.92E-03 | -0.0314 | 0.0110 | 4.18E-03 | 0.0310 | 14 |
| IL7R | 5 | ENSG00000168685 | 35866251 | rs10058453 | 5 | 35880820 | C | T | 0.296524 | 0.2915 | 0.0084 | 4.14E-266 | 105.79 | 0.0066 | 0.0023 | 4.67E-03 | 0.0226 | 0.0080 | 4.81E-03 | 0.0231 | 20 |
| IL18RAP | 2 | ENSG00000115607 | 103052087 | rs6734762 | 2 | 103062926 | T | C | 0.52863 | 0.7870 | 0.0066 | 0.00E+00 | 321.08 | 0.0043 | 0.0022 | 4.42E-02 | 0.0055 | 0.0027 | 4.42E-02 | 0.6309 | 20 |

Supplementary Table 9. Association between eQTL top SNP of FPR1 with expression of other nearby genes (*p*<0.05).

| **Gene** | **Gene Chromosome** | **Gene position (hg19)** | **eQTL SNP** | **SNP Chromosome** | **SNP position (hg19)** | **Effect allele** | **Other allele** | **Effect allele frequency** | **eQTL association** | | |
| --- | --- | --- | --- | --- | --- | --- | --- | --- | --- | --- | --- |
|  |  |  |  |  |  |  |  |  | **beta** | **se** | ***p*-value** |
| FPR3 | 19 | 52258019 | rs6509570 | 19 | 52313929 | T | C | 0.375256 | 0.0099852 | 0.0108344 | 0.3567291 |
| FPR2 | 19 | 52258019 | rs6509570 | 19 | 52264529 | G | T | 0.41002 | 0.0003986 | 0.0031243 | 8.98E-01 |
| LINC00085 | 19 | 52258019 | rs6509570 | 19 | 52206116 | A | G | 0.182004 | 0.0148832 | 0.0160753 | 3.55E-01 |
| SIGLEC14 | 19 | 52258019 | rs6509570 | 19 | 52147930 | T | G | 0.115542 | -0.003414 | 0.0033129 | 3.03E-01 |
| ZNF175 | 19 | 52258019 | rs6509570 | 19 | 52083771 | A | G | 0.246421 | 0.00731 | 0.0062492 | 2.42E-01 |

*No eQTLs for IL1RAP or IL7R are available at a genome-wide significance level (p < 5.0 × 10^-8^).

Supplementary Table 10. IVW-MR association between inflammatory factory mediated by gene FPR1, IL1RAP, IL7R, or IL18RAP and repeat AD GWAS.

| **Exposure** | **Outcome** | **Method** | **Number of SNPs** | **beta** | **se** | **OR** | **95%CI** | **p-value** | **p value for Cochran Q test** | **p value for MR-Egger intercept** | **p value for MR-PRESSO Global test** |
| --- | --- | --- | --- | --- | --- | --- | --- | --- | --- | --- | --- |
| FPR1 | AD | Inverse variance weighted | 11 | -0.625984 | 0.2289552 | 0.53 | 0.34-0.84 | 0.0062553 | 0.302 | 0.256 | 0.3518 |
| IL1RAP | AD | Inverse variance weighted | 28 | 0.0233242 | 0.0057498 | 1.02 | 1.01-1.03 | 4.98E-05 | 0.997 | 0.393 | 0.9985 |
| IL7R | AD | Inverse variance weighted | 5 | -0.055767 | 0.0213161 | 0.95 | 0.91-0.99 | 8.89E-03 | 0.559 | 0.356 | 0.6474 |
| IL18RAP | AD | Inverse variance weighted | 15 | -0.029699 | 0.0116992 | 0.97 | 0.95-0.99 | 1.11E-02 | 0.061 | 0.832 | 0.0772 |

Supplementary Table 11. IVW-MR association between inflammatory factory mediated by gene FPR1, IL1RAP, IL7R, or IL18RAP and repeat asthma GWAS.

| **Exposure** | **Outcome** | **Method** | **Number of SNPs** | **beta** | **se** | **OR** | **95%CI** | **p value** | **p value for Cochran Q test** | **p value for MR-Egger intercept** | **p value for MR-PRESSO Global test** |
| --- | --- | --- | --- | --- | --- | --- | --- | --- | --- | --- | --- |
| FPR1 | asthma | Inverse variance weighted | 11 | 0.0582311 | 0.0091095 | 1.06 | 1.04-1.08 | 1.63E-10 | 0.989 | 0.687 | 0.9938 |
| IL1RAP | asthma | Inverse variance weighted | 18 | -6.60E-04 | 0.0003006 | 1.00 | 0.998-0.999 | 2.81E-02 | 0.645 | 0.241 | 0.6366 |
| IL7R | asthma | Inverse variance weighted | 4 | 0.0043058 | 0.0011831 | 1.00 | 1.00-1.01 | 2.73E-04 | 0.232 | 0.436 | 0.3468 |
| IL18RAP | asthma | Inverse variance weighted | 16 | 0.0001391 | 0.0003009 | 1.00 | 0.99-1.00 | 6.44E-01 | 0.915 | 0.383 | 0.9300 |

Supplementary Table 12. the LD Score regression intercept, heritability and genetic correlation.

| **Trait** | **mean_chisq** | **lambda_gc** | **intercept** | **intercept_se** | **ratio** | **ratio_se** | **h2_observed** | **h2_observed_se** | **h2_Z** | **h2_p** | **rg** | **rg_se** | **rg_p** |
| --- | --- | --- | --- | --- | --- | --- | --- | --- | --- | --- | --- | --- | --- |
| Asthma | 1.11921 | 1.091404 | 1.017881 | 0.008536 | 0.149997525 | 0.07160696 | 0.080591616 | 0.011408062 | 7.064443938 | 1.61E-12 | 0.043579 | 0.081275 | 0.591824 |
| Alzheimer's_disease | 1.118815 | 1.086292 | 1.025042 | 0.02337 | 0.210767136 | 0.196692722 | 0.010042747 | 0.001810899 | 5.545725592 | 2.93E-08 |  |  |  |
